# Supplementary material for: Paranormal beliefs and cognitive function: A systematic review and assessment of study quality across four decades of research
Source: PLoS One. 2022 May 4;17(5):e0267360. doi: 10.1371/journal.pone.0267360 (PMC9067702; doi:10.1371/journal.pone.0267360)
Supplement: S1 Table — Note: Ts = Thinking Style, CPb = Cognitive and Perceptual Biases, O = Other Cognitive Functions, REI = Rational and Experiential Inventory (Epstein et al., 1996), SJQ = Scenario Judgements Questionnaire (Rogers et al., 2016; Rogers et al., 2011), IPO-RT = Inventory of Personality Organization (Lenzenweger et al., 2001), RT = reality testing, ASGS = Australian Sheep-Goat Scale (Thalbourne & Delin, 1993), ESP = extrasensory perception, LAD = life after death, PK = psychokinesis, NAP = new age philosophy, TPB = traditional paranormal beliefs, RPBS = Revised Paranormal Belief Scale (Tobacyk, 2004; Lange et al., 2000), CKCS = Core Knowledge Confusions scale (Lindeman & Aarnio, 2007; Lindeman et al., 2008), CRT = Cognitive Reflection Test (Frederick, 2005), BRC = base-rate conflict, BRN = base-rate neutral, SREIT = Self-Report Emotional Intelligence Test (Schutte et al., 1998), WCQ = Ways of Coping Questionnaire (Folkman & Lazarus, 1988), IBI = Irrational Beliefs Inventory (Koopmans et al., 1994). (DOCX) [file pone.0267360.s003.docx]

**S1 Table. Papers excluded from the review (participants < 18).**

| **Study** | **Sample Size (% women)** | **Age Range, *M* (SD)** | **Area** | **Tests Used** | **Key Findings** |
| --- | --- | --- | --- | --- | --- |
| Rogers et al. (2018) | 261 (54.0) | 16-84, 37.10 (16.50) | Ts | REI, SJQ | - corr. analytical thinking and: ESP (*r* = -.24, *p* < .001), PK (*r* = -.29, *p* < .001), and LAD (*r* = -.24, *p* < .001) subscales of ASGS  + corr. intuitive thinking and: ESP (*r* = .30, *p* < .001), PK (*r* = .24, *p* < .001), and LAD (*r* = .34, *p* < .001) subscales of ASGS  + corr. total number of conjunction errors and: ESP (*r* = .24, *p* < .001), PK (*r* = .19, *p* < .001), and LAD (*r* = .22, *p* < .001) subscales of ASGS  Generation of conjunction errors predicted by: ESP (*exp(b)* = 1.32, *p* < .001), PK (*exp(b)* = 3.16, *p* = .003), and LAD (*exp(b)* = 1.27, *p* < .001) subscales of ASGS |
| Dagnall et al. (2017) | 222 (72.0) | 16-63, 30.77 (11.74) | CPb | IPO-RT (RT subscale) | + corr. paranormal belief and RT scores for: total paranormal belief (*r* = .41, *p* < .001), NAP (*r* = .37, *p* < .001), and TPB (*r* = .38, *p* < .001) subscales of the RPBS - believers demonstrated lower RT ability  RT deficits had positive effect on TPB (β = .54, *p* < .001) and NAP (β = .48, *p* < .001) |
| Rogers et al. (2017) | 130 (57.3) | 16-72, 34.40 (13.30) | CPb | SJQ | + corr. PK subscale of ASGS and: total conjunction errors (*r* = .22, *p* < .05), and errors for related constituent pairings (*r* = .19, *p* < .05)  PK predicted the making of conjunction errors (*exp(b)* = 1.26, *p* = .008) |
| Lindeman et al. (2015) | 2789 (65.0) | 15-69, 28.00 (8.87) | CPb | CKCS, teleology statements | + corr. paranormal beliefs and: ontological confusions (*r* = .46, *p* < .05), and teleology (*r* = .31, *p* < .05)  Ontological confusions (*β* = .41) and teleology (*β* = .15) significantly predicted paranormal beliefs |
| Pennycook et al. (2012) | 53 (72.5) | 16-69, 35.04 (12.77) | Ts | CRT, BRC problems, WordSum, BRN problems | - corr. paranormal beliefs and: BRN (*r* = -.13, *p* < .05), CRT (*r* = -.31, *p* < .05), and BRC (*r* = -.23, *p* < .05)  **Ns.** corr. paranormal beliefs and WordSum  Paranormal beliefs negatively predicted analytical cognitive style (*β* = -.20, *p* < .002) |
| Rogers et al. (2011) | 167 (49.7) | 16-71, 32.00 (13.40) | CPb | SJQ | Significant main effect of belief on number of conjunction errors (*F*(1,135) = 6.40, *p* = .013, *partial ƞ^2^* = .05) – believers made more conjunction errors than sceptics |
| Lindeman & Aarnio (2007) | 239 (77.0) | 16-47, 24.20 (/) | Ts | REI, ontological confusion statements | + corr. intuitive thinking and belief in: paranormal agents (*r* = .49, *p* < .001), paranormal abilities (*r* = .51, *p* < .001), luck (*r* = .43, *p* < .001), astrology (*r* = .54, *p* < .001), and feng shui (*r* = .51, *p* < .001)  - corr. analytical thinking and belief in: paranormal agents (*r* = -.21, *p* < .001), paranormal abilities (*r* = -.19, *p* < .01), luck (*r* = -.22, *p* < .001), astrology (*r* = -.28, *p* < .001), and feng shui (*r* = -.27, *p* < .001)  + corr. all six types of ontological confusions and all five paranormal beliefs (all *r*s ≥ .30, *p*s < .001) |
| Rogers et al. (2006) | 253 (38.7) | 17-82, 38.50 (12.50) | O | SREIT, WCQ | - corr. paranormal beliefs and all four SREIT subscales: mood regulation (*r* = -.17, *p* < .01), appraisal of emotions (*r* = -.17, *p* < .01), social skills (*r* = -.15, *p* < .05), utilisation of emotions (*r* = -.16, *p* < .01)  **Ns.** corr. active-cognitive coping and paranormal beliefs  **Ns.** neither active-cognitive coping or SREIT scores predicted paranormal beliefs |
| Lindeman & Aarnio (2006) | 3261 (74.0) | 15-60, 24.00 (4.67) | Ts | REI | Intuitive thinking positively predicted higher-order paranormal beliefs (*β* = 0.25, *p* < .01), while analytical thinking negatively predicted higher-order paranormal beliefs (*β* = -0.17, *p* < .001) |
| Aarnio & Lindeman (2005) | 3141 (74.0) | 16-60, 24.00 (4.50) | Ts | REI | + corr. paranormal beliefs and intuitive thinking (*r* = .34, *p* < .001)  - corr. paranormal beliefs and analytical thinking (*r* = -.14, *p* < .001) |
| Saher & Lindeman (2005) | 3261 (74.0) | 15-60, 24.00 (/) | Ts | REI | + corr. paranormal beliefs and intuitive thinking (*r* = .34, *p* < .001)  - corr. paranormal beliefs and analytical thinking (*r* = -.15, *p* < .001) |
| Farias et al. (2005) | 99 (56.6) | 17-79, 38.20 (21.10) | CPb | Visual perception task | + corr. paranormal beliefs and complex dot patterns for: total complex reports (*r* = .29, *p* < .01), number of different complex reports (*r* = .25, *p* < .01)  - corr. paranormal beliefs and complex dot patterns for: latency of first complex report (*r* - .24, *p* < .05)  **Ns.** corr. paranormal beliefs and simple dot patterns for: total simple reports, number of different simple reports, or latency of first simple report |
| Roig et al. (1998) | 814 (54.8) | 17-47, 20.40 (/) | Ts | IBI | Paranormal believers scored significantly higher compared to sceptics for: global irrational thinking (*F*(1,407) = 18.24, *p* < .001), rigidity (*F*(1,407) = 15.38, *p* < .001), and worrying (*F*(1,407) = 18.24, *p* < .001) |

*Note: Ts = Thinking Style, CPb = Cognitive and Perceptual Biases, O = Other Cognitive Functions, REI = Rational and Experiential Inventory (Epstein et al., 1996), SJQ = Scenario Judgements Questionnaire (Rogers et al., 2016; Rogers et al., 2011), IPO-RT = Inventory of Personality Organization (Lenzenweger et al., 2001), RT = reality testing, ASGS = Australian Sheep-Goat Scale (Thalbourne & Delin, 1993), ESP = extrasensory perception, LAD = life after death, PK = psychokinesis, NAP = new age philosophy, TPB = traditional paranormal beliefs, RPBS = Revised Paranormal Belief Scale (Tobacyk, 2004; Lange et al., 2000), CKCS = Core Knowledge Confusions scale (Lindeman & Aarnio, 2007; Lindeman et al., 2008), CRT = Cognitive Reflection Test (Frederick, 2005), BRC = base-rate conflict, BRN = base-rate neutral, SREIT = Self-Report Emotional Intelligence Test (Schutte et al., 1998), WCQ = Ways of Coping Questionnaire (Folkman & Lazarus, 1988), IBI = Irrational Beliefs Inventory (Koopmans et al., 1994)*
