# Supplementary material for: Paranormal beliefs and cognitive function: A systematic review and assessment of study quality across four decades of research
Source: PLoS One. 2022 May 4;17(5):e0267360. doi: 10.1371/journal.pone.0267360 (PMC9067702; doi:10.1371/journal.pone.0267360)
Supplement: S2 Table — Note: / = information not reported, P = perceptual biases, C = cognitive biases, bl = believers, sc = sceptics, + = positive,— = negative, corr. = correlation, Ns. = nonsignificant, ESP = extrasensory perception, BADE = bias against disconfirmatory evidence, BACE = bias against confirmatory evidence, TRB = traditional religious beliefs, ELF = extraordinary lifeforms, PRI = Personal Risk Inventory (Hockey et al., 2000), SFQ = Strange-Face Questionnaire (Caputo, 2015), IDAQ = Individual Differences in Anthropomorphism Quotient (Waytz et al., 2010), DS = Dualism Scale (Stanovich, 1989), EQ = Empathy Quotient (Baron-Cohen & Wheelwright, 2004). (DOCX) [file pone.0267360.s004.docx]

**S2 Table. Studies included in the systematic review concerning perceptual and cognitive biases**

| **Study** | **Sample Size (% women)** | **Age Range, *M* (SD)** | **Bias** | **Tests Used** | **Key Findings** |
| --- | --- | --- | --- | --- | --- |
| Lesaffre et al. (2020) | 419 (69.4) | 18-47, 20.50 (3.07) | C | Novel event explanation questionnaire, mental dice task | **Ns.** corr. paranormal beliefs and repetition avoidance  + corr. paranormal beliefs and confirmation bias (*r* = .42, *p* < .001) – higher paranormal belief scores associated with explaining a magic performance as psychic |
| Drinkwater et al. (2019) | 174 (75.9) | 18-62, 24.62(/) | P | PRI | Paranormal belief predicts risk perception (*b* = .059, *t*=3.902, *p* < .001) |
| Griffiths et al. (2019) | 160 (96.2) | /, 19.00(/) | P | Causal judgement task | + corr. paranormal belief and causal judgement ratings (*r*(151) = .22, *p* =.005, *BF* = 4.76) |
| Barberia et al. (2018) | 106 (81.1%) | *intervention* /, 21.57 (3.48)  *control* /, 20.83 (2.65) | C | Novel confirmatory bias education intervention | - effect of intervention on precognition scores (*t*(102) = -2.62, *p* = .005, *d* = -0.52)  **Ns.** effect of intervention on global paranormal belief, witchcraft, TRB, spiritualism, ELF, extra-terrestrial life and actual visits, or superstition scores (*p*s > .26) |
| Prike et al. (2018) | 259 (58.9) | 18-81, 35.44 (11.94) | C | BADE, BACE, liberal acceptance scenarios, jumping to conclusions task | - corr. paranormal belief and BADE (*r* = -.22, *p* < .001), BACE (*r* = -.22, *p* < .001) and + corr. with liberal acceptance (*r* = .35, *p* < .001)  Liberal acceptance predicted paranormal belief (*F*(1, 219) = 9.03, *p* = .003, η_p_^2^ = 0.04, *BF*10 = 12.91) |
| Caputo (2017) | 30 (80.0) | 20-26, 21.70 (1.23) | P | SFQ | **Ns.** corr. overall paranormal belief and strange face illusions, but + corr. paranormal beliefs and two SFQ items (#7, *r* = -.46, *p* < .01; #12, *r* = -.37, *p* < .05) |
| Van Elk (2017) | 53 (77.4) | /, 40.10 (15.40) | P | Computerised card guessing game | **Ns.** relationship paranormal belief and illusion of control (*F*s < 1) |
| Van Elk (2015) | 55 (69.1) | /, 43.40 (/) | P | Novel face/house categorization task | Paranormal belief predicted accuracy on categorisation trials with 70% visual noise (β = 0.353, *p* = .011) |
| Blanco et al. (2015) | 64 (81.3) | 18-26, 18.69 (1.45) | P | Contingency task | + corr. paranormal belief and illusion of control (*r* = .28, *p* < .05)  + effect paranormal belief on illusory control (β = .28, *p* = .02) for noncontingent task |
| Irwin et al. (2014) | 124 (79.0) | 18-65, 26.44 (10.43) | C | Computerised beads task, JTC subscale of CBQ, JTC subscale of DACOBS | - corr. TPB subscale of RPBS and both the 85:15 beads task (*r* = -.22, *p* < .05), and the 60:40 beads task (*r* = -.26, *p* < .01)  + corr. TPB and CBQ (*r* = .23, *p* < .05)  + corr. NAP subscale and DACOBS (*r* = .23, *p* < .01)  **Ns.** corr. NAP and either: 85:15 beads task, 60:40 beads task, CBQ  **Ns.** corr. TPB and DACOBS |
| Simmonds-Moore (2014) | 95 (51.6) | 20-76, 45.00 (13.10) | P | Visual and auditory detection task, each including one ESP, two degraded stimuli and one random trial | Faster response latencies for first guesses in visual degraded stimuli condition for paranormal believers compared to sceptics (*X^2^* = 5.44, df = 2, *p* = .036)  More misidentifications of visual degraded stimuli for believers compared to sceptics (*X^2^* = 7.01, df = 2, *p* = .013)  Believers more confident in first guesses for both visual (*X^2^* = 6.1, df = 2, *p* = .024) and auditory (*X^2^* = 6.2, df = 2, *p* = .022) degraded stimuli |
| Willard & Norenzayan (2013) | 479 (77.0)  825 (66.0) | 18-41, 20.5 (/)  18-81, 34.7 (/) | C | DS, IDAQ, EQ, teleology statements | + corr. paranormal belief and dualism (*r* = .43, *p* ≤ .01), teleology (*r* = .18, *p* ≤ .01), and anthropomorphism (*r* = .36, *p* ≤ .01) in sample 1  + corr. paranormal belief and dualism (*r* = .33, *p* ≤ .01), teleology (*r* = .19, *p* ≤ .01), anthropomorphism (*r* = .31, *p* ≤ .01), and mentalizing (*r* = .12, *p* ≤ .01) in sample 2  + relationship paranormal belief and anthropomorphism (β = .29), dualism (β = .38), and teleology (β = .12) in sample 1  + relationship paranormal belief and anthropomorphism (β = .28), dualism (β = .29), and teleology (β = .05) in sample 2 |
| Riekki et al. (2013) | 47 (55.3) | 20-50, 31.00 (/) | P | Novel face detection task including artefact face pictures vs non-face pictures | Paranormal believers more false alarms for non-face pictures compared to sceptics (*F*(1, 36) = 7.95, *p* = .008, *η_p_^2^* = .181)  Paranormal believers more hits for artefact face pictures compared to sceptics (*F*(1, 36) = 9.99, *p* = .003,  *η_p_^2^* = .217)  Paranormal believers lower response criteria (*F*(1, 36) = 11.02, *p* = .002,  *η_p_^2^* = .234) and higher correct detections (*F*(1, 36) = 6.01, *p* = .019,  *η_p_^2^* = 143) compared to sceptics  Paranormal believers rated artefact faces as more face-like (*F*(1, 37) = 6.25, *p* = .017,  *η_p_^2^* = .145) and emotive (*F*(1, 37) = 4.70, *p* = .037,  *η_p_^2^* = .113) compared to sceptics |
| Van Elk (2013) | 67 (71.6) | /, 28.30 (/) | P | Point-light-walker displays | + corr. paranormal belief score and response bias for stimuli with 12 (*r* = .45, *p* < .001), 24 (*r* = .29, *p* < .05), 48 (*r* = .43, *p* < .001), and 96 distractors (*r* =.41, *p* < .001)  **Ns.** corr. paranormal belief score and stimuli with 192 (*p* = .25) or 384 (*p* < .621) distractors  Difference between sceptics and believers most pronounced for stimuli with low to intermediate number of visual distractors (*F*(5, 290) = 3.1, *p* < .01,  *η_p_^2^* = .05)  Main effect of group on perceptual sensitivity (*F*(1, 58) = 9.5, *p* < .005,  *η_p_^2^* = .14) with sceptics showing higher perceptual sensitivity compared to believers  Main effect of group on bias for reporting illusory patterns (*F*(1, 58) = 8.4, *p* < .005,  *η_p_^2^* = .13) with sceptics showing reduced response bias compared to believers |
| Krummenacher et al. (2010) | 40 (00.0) | 21-39, *bl*28.7 (4.8), *sc*28.4 (4.5) | P | Lexical and facial decision tasks | Main effect of group on response criterion (*F*(1, 35) = 11.12, *p* = .002), with believers demonstrating lower response criteria compared to sceptics  + corr. paranormal belief and schizotypy (*rho* = .92, *p* < .000) |
| Rudski (2004) | 275 (61.5) | 18-25, / (/) | P | Illusion of control questionnaire | Higher paranormal belief scores for participants demonstrating illusion of control (*t*(269) = 2.31, *p* = .02) Higher scores on the superstition (*t*(269) = 4.07, *p* < .001) and precognition (*t*(269) = 3.34, *p* = .001) subscales of the RPBS for participants demonstrating illusion of control |
| Schienle et al. (1996) | 42 (54.8) | 18-29, 23.10 (/) | P | Telepathy experiment | Paranormal believers gave higher estimates of success than sceptics (*F*(1, 38) = 18.6, *p* < .001) Difference between paranormal believers and sceptics in judgement accuracy (*t*(20) = 3.19, *p* = .003), with believers overestimating the number of total hits and sceptics judging the hit rate accurately |
| Blackmore & Moore (1994) | 30 (33.3) | /, / (/) | P | Visual detection task and false identification question | + corr. paranormal belief and false identification question responses, with believers claiming to make more false identifications of people in their daily lives (*r* = .606, *p* < .001)  **Ns.** corr. paranormal belief and response type in visual detection task |
| Gagné & McKelvie (1990) | 53 (37.7) | /, / (/) | P | Signal detection task and questionnaire | **Ns.** difference paranormal believers and sceptics in either the behavioural task or questionnaire (*p*s > .05) |

*Note: / = information not reported, P = perceptual biases, C = cognitive biases, bl = believers, sc = sceptics, + = positive, - = negative, corr. = correlation,* ***Ns.*** *= nonsignificant, ESP = extrasensory perception, BADE = bias against disconfirmatory evidence, BACE = bias against confirmatory evidence, TRB = traditional religious beliefs, ELF = extraordinary lifeforms, PRI = Personal Risk Inventory (Hockey et al., 2000), SFQ = Strange-Face Questionnaire (Caputo, 2015), IDAQ = Individual Differences in Anthropomorphism Quotient (Waytz et al., 2010), DS = Dualism Scale (Stanovich, 1989), EQ = Empathy Quotient (Baron-Cohen & Wheelwright, 2004)*
