# Supplementary material for: Paranormal beliefs and cognitive function: A systematic review and assessment of study quality across four decades of research
Source: PLoS One. 2022 May 4;17(5):e0267360. doi: 10.1371/journal.pone.0267360 (PMC9067702; doi:10.1371/journal.pone.0267360)
Supplement: S3 Table — Note: / = information not reported, + = positive,— = negative, corr. = correlation, Ns. = nonsignificant, ESP = extrasensory perception, PK = psychokinesis, LAD = life after death, NAP = new age philosophy, DR = deductive reasoning, RTQ = Reasoning Task Questionnaire (Blackmore & Troscianko, 1985), ASGS = Australian Sheep-Goat Scale (Thalbourne & Delin, 1993), RPBS = Revised Paranormal Belief Scale (Tobacyk, 2004), MMU-N = Manchester Metropolitan University New (Dagnall et al., 2010). (DOCX) [file pone.0267360.s005.docx]

**S3 Table. Studies included in the systematic review concerning reasoning.**

| **Study** | **Sample Size (% women)** | **Age Range and *M* (SD)** | **Tests Used** | **Key Significant Findings** |
| --- | --- | --- | --- | --- |
| Pérez-Navarro & Martínez-Guerra (2020) | 180 (71.1) | 18-49, 23.20 (5.60) | RTQ | + corr. paranormal belief and schizotypy (*r* =.28, p < .001)  + corr. paranormal belief and conditional reasoning on tasks with paranormal content (*r* = .24, *p* < .001)  **Ns.** corr. paranormal belief and probabilistic reasoning |
| Denovan et al. (2018) | 725 (73.0) | 18-64, 25.50 (9.40) | Perception of randomness, conjunction fallacy, paranormal perception of randomness, and paranormal conjunction fallacy tasks | + corr. paranormal belief and schizotypy (*r* = .32, *p* < .001)  - corr. paranormal belief and perception of randomness (*r* = -.24, *p* < .001) and conjunction fallacy (*r* = -.14, *p* < .001)  - corr. paranormal belief and paranormal perception of randomness (*r* = -.37, *p* < .001) and paranormal conjunction fallacy (*r* = -.45, *p* < .001) |
| Prike et al. (2017) | 434 (/) | /, / (/) | Scenario judgements questionnaire | + corr. between paranormal belief and single conjunction errors in Experiment 1A (*r* = .21, *p* < .001) and 1B (*r* = .31, *p* < .001)  + corr. paranormal belief and conjunction errors in Experiment 1A (*r* = .25, *p* < .001) and 1B (*r* = .33, *p* < .001) |
| Dagnall et al. (2016A) | 254 (73.0) | 18-71, 26.66 (9.81) | Perception of randomness, conjunction fallacy, paranormal perception of randomness, and paranormal conjunction fallacy tasks | + corr. paranormal belief and schizotypy scores (*r* = .25, *p* < .01)  - corr. paranormal belief and perception of randomness (*r* = -.25, *p* < .01), conjunction fallacy (*r* = -.17, *p* < .01), paranormal perception of randomness (*r* = -.38, *p* < .01), and paranormal conjunction fallacy (*r* = -.49, *p* < .01)  - effect of traditional paranormal belief on perception of randomness (β = -.39, *p* < .001) and conjunction fallacy (β = -.25, *p* < .05)  - effect of new age philosophy on perception of randomness (β = -.31, p < .001)  + effect of the unusual experiences O-LIFE subscale on the traditional paranormal belief (β = .31, p < .001) and new age philosophy (β = .31, p < .001)  **Ns.** effect of NAP on conjunction fallacy |
| Dagnall et al. (2016B) | 233 (74.0) | 18-65, 23.00 (8.41) | Perception of randomness, conjunction fallacy, paranormal perception of randomness, and paranormal conjunction fallacy tasks | - corr. paranormal belief and perception of randomness (*r* = -.25, *p* < .01) and conjunction fallacy (*r* = -.12, *p* < .05)  - corr. paranormal belief and paranormal perception of randomness (*r* = -.43, *p* < .01) and paranormal conjunction fallacy (*r* = -.46, *p* < .01) |
| Rogers et al. (2016) | 213 (42.2) | 18-74, 29.40 (10.40) | Scenario Judgements Questionnaire | + corr. paranormal belief subscales and conjunction errors for paranormal and non-paranormal scenarios with confirmatory outcomes: ESP (*r* = .25, *p* < .001), PK (*r* = .30, *p* < .001), and LAD (*r* = .21, *p* < .01) subscales  + corr. paranormal belief subscales and conjunction error rates for non-paranormal scenarios with confirmatory outcomes: ESP (*r* = .21, *p* < .01), PK (*r* = .30, *p* < .001), LAD (*r* = .19, *p* < .01) subscales  + corr. paranormal scenarios with confirmatory outcomes: ESP (*r* = .18, *p* < .05), PK (*r* = .19, *p* < .01), LAD (*r* = .15, p < .05) subscales  Paranormal belief predicted number of conjunction errors: ESP (β = .18, p = .001), PK (β = .19, p < .001), LAD (β = .14, p = .004) subscales |
| Dagnall et al. (2014) | 305 (79.0) | 18-65, 22.97 (8.19) | Perception of randomness, conjunction fallacy, paranormal conjunction fallacy, probability, and base rate tasks | - corr. overall probabilistic reasoning and paranormal belief on three scales - ASGS (*r* = -.19, *p* < .01), MMU-N (*r* = -.23, *p* < .01), and RPBS (*r* = -.17, *p* < .01) Perception of randomness predicted paranormal belief for the ASGS (β = -.17, *p* = .04), MMU-N (β = -.16, *p* = .005), and RPBS (β = -.16, *p* = .008)  - corr. base rate test scores and ASGS (*r* = -.12, *p* < .05), MMU-N (*r* = -.13, *p* < .05), and RPBS (*r* = -.07, *p* < .05) - corr. probability test scores and ASGS (*r* = -.14, *p* < .01), MMU-N (*r* = -.14, *p* < .01), and RPBS (*r* = -.10, *p* < .05) - corr. perception of randomness and ASGS (*r* = -.18, *p* < .01), MMU-N (*r* = -.19, *p* < .01), and RPBS (*r* = -.17, *p* < .01)  **Ns.** corr. paranormal belief and conjunction fallacy for ASGS, MMU-N and RPBS |
| Rogers et al. (2009) | 200 (59.0) | 18-56, 22.20 (5.30) | Scenario judgements questionnaire | Paranormal believers made more conjunction errors overall compared to sceptics (*F*(1, 195) = 53.89, *p* < .001)  Sceptics made fewer conjunction errors for paranormal events compared to non-paranormal events (*F*(1, 96) = 32.73, *p* < .001)  Believers made fewer conjunction errors for paranormal events compared to non-paranormal events (*F*(1, 100) = 22.19, *p* < .001)  Believers made more conjunction errors compared to sceptics for both the probability (*F*(1, 97) = 43.77, *p* < .001) and frequency (*F*(1, 97) = 16.10, *p* < .001) questions |
| Dagnall et al. (2007) | 96 (77.1) | 18-47, 20.39 (4.85) | Perception of randomness, conjunction fallacy, base rate, and probability tasks | - corr. paranormal belief and overall number of correct responses (*r* = -.24, *p* = .018)  Perception of randomness scores predicted paranormal belief (β = -.32, *p* = .002)  - corr. perception of randomness and paranormal belief (*r* = -.32, *p* = .001)  Participants low in paranormal belief solved more perception of randomness questions than those high in paranormal belief (*t*(94) = 2.50, *p* = .014)  **Ns.** difference between high and low believers for base rate, probability, or conjunction fallacy questions (*p* > .05) |
| Lawrence & Peters (2004) | 174 (27.6) | 18-86, 54.0 (/) | DR task | Difference in number of overall DR errors (*U* = 2691.5, *p* = .002) with strong believers making more errors than weak believers  Strong believers made more errors on both the control (*U* = 2925.5, *p* = .01) and antiparanormal (*U* = 2668.5, *p* = .001) statements  **Ns.** difference in number of errors for proparanormal statements between strong and weak believers (*p* = .10) |
| Musch & Ehrenberg (2002) | 123 (56.1) | /, 24.70 (5.60) | Probabilistic test battery | + corr. paranormal belief and probabilistic reasoning errors (*r* = .22, *p* < .05)  + corr. paranormal belief and lower cognitive ability (*r* = .50, *p* < .01)  + corr. paranormal belief and lower cognitive ability when controlling for probabilistic reasoning skills (*r* = .47, *p* < .01) |
| Bressan (2002) | 111 (65.8)  103 (48.5) | 19-62, / (/)  19-61, / (/) | Probabilistic reasoning questionnaire | - corr. paranormal belief and probabilistic tasks for representativeness bias to sample size (*rho* = -.38, *p* < .001), and representativeness applied to random sequences (*rho* = -.27, *p* = .006) in sample two  **Ns.** difference in probabilistic reasoning scores between believers and sceptics in sample one |
| Roberts & Seager (1999) | 65 (63.1) | 18-75, 33.70 (11.30) | Probabilistic reasoning and conditional reasoning tasks | - corr. paranormal belief and reasoning ability (*r* = -.25, *p* < .05)  **Ns.** corr. paranormal belief and probabilistic reasoning (*p* > .05) - corr. overall conditional reasoning score and paranormal belief (*r* = -.27, *p* < .05) |
| Blackmore (1997) | 6238 (59.0) | /, / (/) | Probabilistic reasoning questionnaire | Believers claimed more of the statements were true for them (*M* = 2.56) compared to sceptics (*M* = 2.23), and these differences were significant for 5 out of 10 items  **Ns.** difference in probability misjudgements between believers and sceptics |
| Brugger et al. (1991) | 95 (/) | /, / (/) | Probability judgement task | Believers demonstrated higher illusion of control and probabilistic reasoning errors compared to sceptics (*χ^2^* = 3.92, *p* = .023) |
| Brugger et al. (1990) | 48 (50.0) | 20-40, / (/) | Mental dice task | Believers made fewer repetitions compared to sceptics (*t* = 1.8, *p* < .05), and made increased errors when making probability judgements (*t* = 2.8, *p* < .005) |
| Wierzbicki (1985) | 64 (53.1%) | /, / (/) | Conditional reasoning statements | + relationship between paranormal belief scores and reasoning errors (*F*(1, 63) = 6.37, *p* < .05) + corr. paranormal belief scores and total number of reasoning errors (*r* = .28, *p* < .05) **Ns.** interaction between paranormal belief and logical form **Ns.** interaction between paranormal belief and content (symbolic and parapsychological problems) |

*Note: / = information not reported, + = positive, - = negative, corr. = correlation,* ***Ns.*** *= nonsignificant, ESP = extrasensory perception, PK = psychokinesis, LAD = life after death, NAP = new age philosophy, DR = deductive reasoning, RTQ = Reasoning Task Questionnaire (Blackmore & Troscianko, 1985), ASGS = Australian Sheep-Goat Scale (Thalbourne & Delin, 1993), RPBS = Revised Paranormal Belief Scale (Tobacyk, 2004), MMU-N = Manchester Metropolitan University New (Dagnall et al., 2010)*
