# Supplementary material for: Paranormal beliefs and cognitive function: A systematic review and assessment of study quality across four decades of research
Source: PLoS One. 2022 May 4;17(5):e0267360. doi: 10.1371/journal.pone.0267360 (PMC9067702; doi:10.1371/journal.pone.0267360)
Supplement: S4 Table — Note: / = information not reported, C = cognitive ability, I = intelligence, m = males, f = females, + = positive,— = negative, corr. = correlation, Ns. = nonsignificant, ATS = Assessment of Thinking Skills (Wesp & Montgomery, 1998), WGCTA-S = Watson-Glaser Critical Thinking Appraisal Form S (Watson & Glaser, 1994), WGCTA = Watson-Glaser Critical Thinking Appraisal (Watson & Glaser, 2002; Watson & Glaser, 1980; Watson & Glaser, 1964), RPM = Raven’s Progressive Matrices (Raven et al., 2000), RPM Rasch Model = Raven’s Progressive Matrices Rasch Model (Rasch, 1960), MHVT = Mill Hill Vocabulary Test (Raven et al., 1998), CCTT = Cornell Critical Thinking Test (Ennis & Millman, 1985), WMT = Wiener Matrizen Test (Formann & Piswanger, 1979), APM = Advanced Progressive Matrices (Raven, 1976), WAIS-IS = Wechsler Adult Intelligence Scale Information Subtest (Wechsler, 1955), GPA = Grade Point Average. (DOCX) [file pone.0267360.s006.docx]

**S4 Table. Studies included in the systematic review concerning intelligence, critical thinking, and academic performance.**

| **Study** | **Sample Size (% women)** | | **Age Range and *M* (SD)** | **Focus of Study** | **Tests Used** | **Key Significant Findings** |
| --- | --- | --- | --- | --- | --- | --- |
| Betsch et al. (2020) | 599 (60.0) | 18-81, 33.63 (11.38) | | I | IQ | Paranormal beliefs negatively predicted by IQ when sex is excluded from the model (*β* = -.158, *p* < .001) |
| Andrews & Tyson (2019) | 687 (54.4) | 18-65, 24.62 (/) | | C | Average university assignment grade | - corr. mean grade and paranormal beliefs (*r*(162) = -0.388, *p* = .001), higher paranormal beliefs ‘soft science’ and ‘artistic’ students than ‘hard science’ students |
| Wilson (2018) | 340 (52.7) | /, / (/) | | C | Science and critical thinking course | Belief in psychics (*T_309_* = 3.14, *p* < .001), witchcraft (*T_308_* = 5.68, *p* < .001), spiritualism (*T_309_*) = 9.23, *p* < .001), monsters ( *T_309_*) = 9.13, *p* < .001), precognition (*T_308_* = 8.53, *p* < .001), aliens (*T_310_* = 13.49, *p* < .001), and alternative medicine (*T_310_* = 15.83, *p* < .001) lowered following critical thinking course  **Ns.** difference in superstition following critical thinking course |
| McLean & Miller (2010) | 47 (70.2) | /, 21.33 (1.74) | | C | ATS, WGCTA-S, course in critical thinking skills | Paranormal belief decreased following critical thinking course (*F*(1, 44) = 48.71, *p* < .001)  Main effect of time, lower levels of paranormal belief post-test compared to pre-test (*F*(1, 44) = 44.63, *p* < .001) |
| Stuart-Hamilton et al. (2006) | 73 (/) | 60-84, 71.12 (5.21) | | I | RPM, MHVT, and probability tests | **Ns.** corr. paranormal belief and intelligence (fluid or crystallised), paranormal belief and probability tests |
| Hergovich & Arendasy (2005) | 180 (59.4) | 18-37, 24.54 (3.61) | | C, I | CCTT, WGCTA, WMT, RPM Rasch Model | - corr. reasoning ability and traditional paranormal belief (*r* = -.22, *p* < .01), superstition (*r* = -.23, *p* < .01), and traditional religiosity (*r* = -.23, *p* < .01)  Univariate effect of reasoning ability for traditional paranormal belief (*F*(1, 166) = 8.62, *p* < .01) and new age philosophy (*F*(1, 166) = 6.35, *p* < .05)  **Ns.** effect of critical thinking ability on paranormal belief (*p* > .05) |
| Roe (1999) | 117 (65.8) | /, 21.00 (/) | | C | Evaluation of an experimental report | **Ns.** main effects for paranormal belief and critical thinking ability |
| Morgan & Morgan (1998) | 124 (65.0) | 18-54, 24.30 (/) | | C | WGCT | - corr. Superstitious Belief subscale and overall WGCT score (*r* = -.19, *p* < .05), as well as with the Inference subscale of the WGCT (*r* = -.17, *p* < .05)  - corr. Traditional Religious Belief subscale and Evaluation of Arguments subscale of the WCGT (*r* = -.18, *p* < .05)  - corr. Spiritualism subscale and the Inference (*r* = -.21, *p* < .05) and Recognition of Assumptions (*r* = -.18, *p* < .05) subscales of the WGCT  **Ns.** corr. full scale scores for paranormal belief and critical thinking |
| Smith et al. (1998) | 60 (40.0) | 18-37, 21.10 (/) | | I | APM Set 1 | - corr. overall paranormal belief and intelligence (*rho* = -.29, *p* < .01)  - corr. intelligence and spiritualism (*rho* = -.35, *p* < .01), psi (*rho* = -.30, *p* < .01), and precognition (*rho* = -.25, *p* < .05) |
| Royalty (1995) | 97 (49.5) | 18-41, 20.5 (/)  18-81, 34.7 (/) | | C | CCTT Level Z, WAIS-IS | **Ns.** corr. paranormal belief and general critical thinking ability, or IQ |
| Tobacyk (1984) | 307 (45.0) | *m* /, 19.70 (1.70)  *f* /, 19.3 (1.7) | | C | GPA | - corr. GPA and Witchcraft (*r* = -.13, *p* < .03) and Superstition (*r* = -.20, *p* < .001) subscales  **Ns.** corr. total paranormal belief and GPA |
| Alcock & Otis (1980) | 26 (/) | /, / (/) | | C | WGCTA | Sceptics demonstrated a higher level of critical thinking ability than believers (*t*(24) = 2.07, *p* < .05) |

*Note: / = information not reported, C = cognitive ability, I = intelligence, m = males, f = females, + = positive, - = negative, corr. = correlation,* ***Ns.*** *= nonsignificant, ATS = Assessment of Thinking Skills (Wesp & Montgomery, 1998), WGCTA-S = Watson-Glaser Critical Thinking Appraisal Form S (Watson & Glaser, 1994), WGCTA = Watson-Glaser Critical Thinking Appraisal (Watson & Glaser, 2002; Watson & Glaser, 1980; Watson & Glaser, 1964), RPM = Raven’s Progressive Matrices (Raven et al., 2000), RPM Rasch Model = Raven’s Progressive Matrices Rasch Model (Rasch, 1960), MHVT = Mill Hill Vocabulary Test (Raven et al., 1998), CCTT = Cornell Critical Thinking Test (Ennis & Millman, 1985), WMT = Wiener Matrizen Test (Formann & Piswanger, 1979), APM = Advanced Progressive Matrices (Raven, 1976), WAIS-IS = Wechsler Adult Intelligence Scale Information Subtest (Wechsler, 1955), GPA = Grade Point Average*
