# Supplementary material for: Paranormal beliefs and cognitive function: A systematic review and assessment of study quality across four decades of research
Source: PLoS One. 2022 May 4;17(5):e0267360. doi: 10.1371/journal.pone.0267360 (PMC9067702; doi:10.1371/journal.pone.0267360)
Supplement: S5 Table — Note: / = information not reported, + = positive,— = negative, corr. = correlation, Ns. = nonsignificant, AOT = Actively Open-Minded Thinking Scale (Stanovich et al., 2016; Stanovich, 1999), CRT = Cognitive Reflection Test (Frederick, 2005), CRT-2 = Cognitive Reflection Test-2 (Thompson & Oppenheimer, 2016), REI = Rational-Experiential Inventory (Pacini & Epstein, 1999), WST = WordSum Test (Huang & Hauser, 1998), RI = Rational/Experiential Inventory (Norris & Epstein, 2011), IPSI-SF = Information-Processing Style Inventory Short Form (Naito et al., 2004), FIS = Faith in Intuition Scale (Pacini & Epstein 1999), NFC = Need for Cognition scale (Cacioppo et al., 1984), AET = Argument Evaluation Test (Stanovich & West, 1997), 10-Item REI = 10-Item Rational-Experiential Inventory (Epstein et al., 1996), GWT = Gestaltwahrnehmungs Test (Hergovich & Hörndler, 1994), EFT = Embedded Figures Test (Witkin et al., 1971). (DOCX) [file pone.0267360.s007.docx]

**S5 Table. Studies included in the systematic review concerning thinking style.**

| **Study** | **Sample Size (% women)** | **Age Range and *M* (SD)** | **Tests Used** | **Key Significant Findings** |
| --- | --- | --- | --- | --- |
| Ballová Mikušková & Čavojová (2020) | 473 (50.5)  492 (53.0) | 18-67, 41.54 (13.75)  18-66, 39.79 (13.68) | CRT | - corr. paranormal beliefs and analytical thinking (*r* = -.149, *p* < .01) in study 1  **Ns.** corr. paranormal beliefs and analytical thinking in study 2 |
| Rizeq et al. (2020) | 321 (81.9) | 18-30, 19.36 (2.09) | AOT, CRT, verbal and non-verbal reasoning tasks | - corr. paranormal belief and cognitive ability (*r* = -.14, *p* < .05), as well as scores on the CRT (*r* = -.22, *p* < .05), and AOT (*r* = -.44, *p* < .05) AOT scored predicted paranormal belief scores (β = -.39, *p* < .001)  **Ns.** cognitive ability and cognitive reflection did not predict paranormal belief |
| Branković (2019) | 257 (43.0) | /, 21.94 (5.74) | REI | Intuitive cognitive style predicted belief in ESP (β = .35, *p* < .001)  Traditional superstitious beliefs best predicted by a lack of rational engagement (β = -.27, *p* = .001) and self-rated intuitive ability (β = .20, *p* = .018) |
| Rogers et al. (2019) | 343 (61.5) | 18-80, 40.50 (12.60) | REI | Partial correlation between intuitive thinking style and paranormal belief when controlling for sampling method (*r* = .57, *p* < .001)  **Ns.** corr. paranormal belief and analytical thinking |
| Ståhl & van Prooijen (2018) | 343 (62.0)  322 (47.0) | /, 35.41 (12.01)  /, 34.95 (10.86) | CRT, CRT-2, Numeracy test, WST | Analytic cognitive style associated with weaker paranormal belief (*b* = -0.44, *SE* = 0.09, *t* = -4.93, *p* < .001) in sample one  Analytic cognitive style associated with weaker paranormal belief (*b* = -0.18, *SE* = 0.05, *t* = -3.81, *p* < .001) in sample two  Cognitive ability contributes to scepticism when controlling for analytic cognitive style for individuals who value epistemic rationality (*b* = -0.38, *SE* = 0.08, *t* = -4.90, *p* < .001)  **Ns.** effect analytic cognitive style on paranormal belief when controlling for cognitive ability |
| Lindeman & Svedholm-Häkkinen (2016) | 258 (63.6) | 18-65, 31.81 (9.89) | Mental rotation test, CRT, RI, maths and physics school grades | - corr. paranormal belief and analytical thinking ability (*r* = -.30, *p* < .001), analytical thinking style (*r* = -.33, *p* < .001), mental rotation (*r* = -.13, *p* < .05), and grades in physics (*r* = -.17, *p* < .01) and maths (*r* = -.16, *p* < .01)  + corr. paranormal belief and intuitive thinking style (*r* = .50, *p* < .001)  Intuitive thinking predicted paranormal beliefs (β = .34, *p* < .001) |
| Lasikiewicz (2016) | 82 (77.0) | 18-62, 29.96 (12.53) | REI | + corr. paranormal belief an intuitive thinking (*r* = .31, *p* < .01)  Paranormal belief predicted by both analytic (β = .246, *p* = .030) and intuitive thinking styles (β = .294, *p* = .018) |
| Irwin (2015) | 94 (58.5) | 18-73, 34.73 (15.19) | REI | + corr. paranormal belief and intuitive thinking (*rho* = .41, *p* < .05)  Intuitive thinking style predicted paranormal belief (*beta* = .39, *p* < .001)  Relationship between paranormal belief and intuitive thinking style (*F*(3, 90) = 6.65, *p* < .001)  **Ns.** relationship paranormal belief and analytical thinking style (*p* = .336) |
| Majima (2015) | 246 (76.4) | 18-81, 25.00 (13.00) | IPSI-SF, logical reasoning task | Intuitive thinking style (β = 0.24, *p* < .001) and analytical thinking style (β = 0.14, *p* = .039) both predicted paranormal belief  **Ns.** relationship paranormal belief and cognitive ability (*p* = .956) |
| Svedholm & Lindeman (2013) | 50 (74.0)  458 (77.1) | 19-62, 34.00 (/)  18-65, 27.00 (7.90) | FIS, NFC, AOT, AET, REI | + corr. paranormal belief and intuitive thinking (*r* = .37, *p* = .01) in sample one  + corr. paranormal belief and inhibition errors (*r* = .22, *p* = .14) in sample one  Paranormal belief predicted by intuitive thinking in sample one (β = .368, *p* = .009) and sample two (β = .460, *p* < .001)  - corr. paranormal belief and AOT scores in sample one (*r* = -.19, *p* = .19) and sample two (*r* = -.41, *p* < .001)  - corr. paranormal belief and NFC scores in sample one (*r* - -.20, *p* = .16) and sample two (*r* = -.21, *p* < .001)  - corr. paranormal belief and AET scores (r = -.30, p = .04) in sample one |
| Genovese (2005) | 96 (71.1) | 20-57, 28.00 (/) | 10-item REI | + corr. paranormal belief and intuitive thinking (*r* = .32, *p* < .01)  + corr. paranormal belief and the cognitive perceptual (*r* = .65, *p* < .01) and disorganized (*r* = .27, *p* < .01) subscales of the SPQ-B  **Ns.** corr. paranormal belief and rational thinking, or between paranormal belief and interpersonal subscale of SPQ-B |
| Hergovich (2003) | 91 (57.1)  150 (57.3) | 18-60, 34.15 (13.98)  /, 37.28 (13.31) | GWT, EFT | - corr. field dependence and paranormal belief (*r* = -.39, *p* < .001) in study one  - corr. field dependence and superstition subscale of PBS (*r* = .45, *p* < .01)  **Ns.** corr. field dependence and total paranormal belief in study two  **Ns.** main effect of field dependence on paranormal belief in study three |
| Gianotti et al. (2001) | 24 (54.2) | /, / (/) | Novel word association task | Interaction effect between belief group and stimulus type (*F*(1, 22) = 6.92, *p* < .015)  Believers produced more rare associations compared to sceptics for unrelated word pairs (19.3% vs 12.0%, *p* < .04)  **Ns.** differences in response latencies for believers and sceptics (*p* = .087) |

*Note: / = information not reported, + = positive, - = negative, corr. = correlation,* ***Ns.*** *= nonsignificant, AOT = Actively Open-Minded Thinking Scale (Stanovich et al., 2016; Stanovich, 1999), CRT = Cognitive Reflection Test (Frederick, 2005), CRT-2 = Cognitive Reflection Test-2 (Thompson & Oppenheimer, 2016), REI = Rational-Experiential Inventory (Pacini & Epstein, 1999), WST = WordSum Test (Huang & Hauser, 1998), RI = Rational/Experiential Inventory (Norris & Epstein, 2011), IPSI-SF = Information-Processing Style Inventory Short Form (Naito et al., 2004), FIS = Faith in Intuition Scale (Pacini & Epstein 1999), NFC = Need for Cognition scale (Cacioppo et al., 1984), AET = Argument Evaluation Test (Stanovich & West, 1997), 10-Item REI = 10-Item Rational-Experiential Inventory (Epstein et al., 1996), GWT = Gestaltwahrnehmungs Test (Hergovich & Hörndler, 1994), EFT = Embedded Figures Test (Witkin et al., 1971)*
