# Supplementary material for: Paranormal beliefs and cognitive function: A systematic review and assessment of study quality across four decades of research
Source: PLoS One. 2022 May 4;17(5):e0267360. doi: 10.1371/journal.pone.0267360 (PMC9067702; doi:10.1371/journal.pone.0267360)
Supplement: S6 Table — Note: / = information not reported, M = memory, EF = executive function, bl = believers, sc = sceptics, + = positive,— = negative, corr. = correlation, Ns. = nonsignificant, DRM = Deese-Roediger-McDermott (Roediger & McDermott, 1995), CRT = Criterial Recollection Task (Gallo, 2013), IIT = Imagination Inflation Task (Garry et al., 1996), RSPAN = Reading-Span Task (Daneman & Carpenter, 1980), OSPAN = Operation Span Task (Turner & Engle, 1989), SILS = Shipley Institute of Living Scale (Zachary, 1986), AET = Argument Evaluation Task (Stanovich & West, 1997), RAT = Remote Associations Test (Mednick, 1962), WCST = Wisconsin Card Sorting Test (Berg, 1948; Grant & Berg, 1948), EFI = Executive Function Index (Spinella, 2005), ANP = anomalous natural phenomena, TRB = traditional religious beliefs, NCQ = News Coverage Questionnaire (Wilson & French, 2006), ASGS = Australian Sheep-Goat Scale (Thalbourne 1995; Thalbourne & Delin, 1993), AEI = Anomalous Experiences Inventory (Kumar et al., 1994). (DOCX) [file pone.0267360.s008.docx]

**S6 Table. Studies included in the systematic review concerning executive function and memory.**

| **Study** | **Sample Size (% women)** | **Age Range and *M* (SD)** | **Focus of Study** | **Tests Used** | **Key Significant Findings** |
| --- | --- | --- | --- | --- | --- |
| Gray & Gallo (2016) | 84 (53.6)  115 (48.7)  95 (48.4) | *bl* /, 27.3 (/)  *sc* /, 26.5 (/)  *bl* /, 27.04 (/)  *sc* /, 27.44 (/)  *bl* /, 27.4 (/)  *sc* /, 27.0 (/) | M | DRM task, CRT, IIT, RSPAN, OSPAN, SILS, AET, RAT | *Study one:*  Sceptics remembered more studied words in DRM task compared to believers for both no warning (*t*(82) = 3.23, *p* < .001) and warning conditions (*t*(82) = 2.88, *p* = .005)  Sceptics better than believers at identifying critical lure as a missing item in DRM task (*t*(82) = 2.54, *p* = .01)  Sceptics recalled fewer critical lures than believers for the warning condition in DRM task (*t*(82) = 2.50, *p* = .01)  Sceptics solved more logic problems on the SILS (*t*(81) = 2.56, *p* = .01), and identified more words in the vocabulary test of the SILS (*t*(81) = 3.17, *p* < .01) than believers  **Ns.** difference in number of false recalls of non-critical words (*p* = .41) or of critical lures (*p* = .74) recalled in no warning condition of the DRM task between sceptics and believers  **Ns.** difference in number of falsely recalled noncritical words between sceptics and believers for warning condition of DRM task (*p* = .24)  **Ns.** difference between believers and sceptics on either the red-word test (*p* =.89), picture test (*p* = .43) or exclusion test (*p* = .15) of the CRT  *Study two:*  Believers remembered more words per list in correct serial position compared to sceptics for RSPAN (*t*(113) = -2.21, *p* = .03)  **Ns.** difference between believers and sceptics for OSPAN (*p* = .16), RAT (*p* = .06) or AET (*p* = .24)  *Study three:*  Sceptics better than believers at identifying critical lure as missing item in the DRM task (*t*(93) = 3.68, *p* < .001)  **Ns.** difference between sceptics and believers for recall of studied words (*p* = .31), false recall of critical lures (*p* = .28) or false recall of non-critical words (*p* = .61) in DRM task  **Ns.** difference between sceptics and believers for RSPAN (*p* = .13), OSPAN (*p* = .66), or for the logic (*p* = .10) and vocabulary (*p* = .09) tests of the SILS  *Studies one and three – pooled data:*  Sceptics solved more of the SILS logic problems (*t*(168) = 3.03, *p* < .01), and identified more words in the vocabulary SILS test (*t*(168) = 3.42, *p* < .01) than believers  *Studies two and three – pooled data:*  Believers remembered more words per list in correct serial position for RSPAN task (*t*(208) = -2.67, *p* = .008) than sceptics  **Ns.** difference between believers and sceptics for OSPAN (*p* = .19), RAT (*p* = .07) or AET (*p* = .06) |
| Lindeman et al. (2011) | 26 (61.5) | *bl* /, 34.6 (/)  *sc* /, 32.2 (/) | EF | Stroop task, WCST | In total, believers and sceptics performed differently on all four subscales of the WCST (*F*(5, 20) = 3.47, *p* =.02, *η^2^* = .398)  Believers’ had higher total errors (*p* < .01), non-perseverative errors (*p* < .01), perseverative errors (*p* < .03), and lower categories correct (*p* < .05) compared to sceptics  **Ns.** difference between believers and sceptics for Stroop |
| Wain & Spinella (2007) | 213 (66.2) | 18-83, 28.0 (11.9) | EF | EFI | - corr. total paranormal beliefs and: overall EF (*r* = -.19, *p* < .01), impulse control (*r* = -.29, *p* < .001), and organisation (*r* = -.23, *p* < .001)  - corr. superstition subscale and: overall EF (*r* = -.30, *p* < .001), motivational drive (*r* = -.17, *p* < .05), empathy (*r* = -.23, *p* < .01), and organisation (*r* = -.33, *p* < .01)  - corr. ANP subscale and: overall EF (*r* = -.19, *p* < .01), impulse control (*r* = -.32, *p* < .001), and organisation (*r* = -.26, *p* < .001)  - corr. psychic beliefs subscale and: impulse control (*r* = -.25, *p* < .001) and organisation (*r* = -.13, *p* < .05)  - corr. witchcraft subscale and impulse control (*r* = -.22, *p* < .01)  - corr. TRB subscale and: motivational drive (*r* = .14, *p* < .05) and empathy (*r* = -.21, *p* < .01)  **Ns.** corr. total paranormal beliefs and: motivational drive, empathy or strategic planning  **Ns.** corr. ANP subscale and either motivational drive, empathy, or strategic planning  **Ns.** corr. psychic beliefs subscale and either motivational drive, empathy, strategic planning, or overall EF  **Ns.** corr. superstition subscale and either impulse control or strategic planning  **Ns.** corr. witchcraft subscale and either motivational drive, empathy, organisation, strategic planning, or overall EF  **Ns.** corr. TRB subscale and either impulse control, organisation, strategic planning, or overall EF |
| Wilson & French (2006) | 100 (58.0) | 23-52, 33.4 (9.87) | M | NCQ | Higher ASGS scores (*t*(98) = 3.49, *p* = .001) and higher scores on the belief subscale of the AEI (*t*(98) = 4.26, *p* < .001) for participants reporting false memories compared to participants not reporting false memories  Paranormal beliefs measured with belief subscale of AEI predicted false memory responses (β = .28, *p* = .04)  **Ns.** ASGS scores did not predict false memory responses |
| Greening (2002) † | 16 (81.3)  52 (78.8)  53 (79.2) | /, 22.4 (4.83)  /, 25.4 (7.66)  /, 56.3 (8.55) | M | False memories questionnaire | + corr. paranormal belief and number of false memories in study 2.1. (*r* = .52, *p* < .05)  **Ns.** corr. paranormal beliefs and number of false memories in studies 2.2 (*p* =.17) and 2.3 (*p* = .62) |
| Dudley (1999) | 56 (64.3) | 18-24, / (/) | M | Digit span test | Higher paranormal belief scores in experimental group (working memory restricted) compared to control group (*F*(1,50) = 5.44, *p* < .05) |

*Note: / = information not reported, M = memory, EF = executive function, bl = believers, sc = sceptics, + = positive, - = negative, corr. = correlation,* ***Ns.*** *= nonsignificant, DRM = Deese-Roediger-McDermott (Roediger & McDermott, 1995), CRT = Criterial Recollection Task (Gallo, 2013), IIT = Imagination Inflation Task (Garry et al., 1996), RSPAN = Reading-Span Task (Daneman & Carpenter, 1980), OSPAN = Operation Span Task (Turner & Engle, 1989), SILS = Shipley Institute of Living Scale (Zachary, 1986), AET = Argument Evaluation Task (Stanovich & West, 1997), RAT = Remote Associations Test (Mednick, 1962), WCST = Wisconsin Card Sorting Test (Berg, 1948; Grant & Berg, 1948), EFI = Executive Function Index (Spinella, 2005), ANP = anomalous natural phenomena, TRB = traditional religious beliefs, NCQ = News Coverage Questionnaire (Wilson & French, 2006), ASGS = Australian Sheep-Goat Scale (Thalbourne 1995; Thalbourne & Delin, 1993), AEI = Anomalous Experiences Inventory (Kumar et al., 1994)*
