# Supplementary material for: Paranormal beliefs and cognitive function: A systematic review and assessment of study quality across four decades of research
Source: PLoS One. 2022 May 4;17(5):e0267360. doi: 10.1371/journal.pone.0267360 (PMC9067702; doi:10.1371/journal.pone.0267360)
Supplement: S7 Table — Note: / = information not reported, bl = believers, sc = sceptics, f = females, m = males, ISL = implicit sequence learning, ISP = implicit semantic priming, VF = visual field, LVF = left visual field, RVF = right visual field, CME = central monitoring efficiency, RE = reasoning errors, CC = cognitive complexity, + = positive,— = negative, corr. = correlation, Ns. = nonsignificant, SPQ-B = Schizotypal Personality Questionnaire Brief (Raine & Benishay, 1995), RCRG = Role Construct Repertory Grid (Kelly, 1955). (DOCX) [file pone.0267360.s009.docx]

**S7 Table. Studies included in the systematic review concerning other cognitive functions.**

| **Study** | **Sample Size (% women)** | | **Age Range and *M* (SD)** | **Focus of Study** | **Tests Used** | **Key Significant Findings** |
| --- | --- | --- | --- | --- | --- | --- |
| Palmer et al. (2007) | 40 (00.0) | 20-40, / (/) | | ISL | Arrow prediction task | Difference in schizotypy scores between believers and sceptics (*t*(33) = 15.1, *p* < .001) with believers scoring higher than sceptics  **Ns.** difference in sequence learning scores between believers and sceptics |
| Pizzagalli et al. (2001) | 24 (100.0) | *bl* /, 26.3 (6.2)  *sc* /, 26.8 (4.3) | | ISP | Semantic priming task | *Word types (prime-target relation):*  Group x VF interaction for indirectly related targets (*F*(1, 22) = 6.32, *p* < .02)  Main effect of group x VF x category (*F*(2, 44) = 5.11, *p* , < .01)  Believers had shorter reaction times for indirectly related target words presented in the LVF compared to sceptics (*p* < .001)  Sceptics shorter reaction times for directly related target words presented in the LVF compared to both indirectly related (900ms ± 90 vs 1081ms ± 209, *p* < .001) and unrelated target words (900ms ± 90 vs 1058ms ±)  Sceptics shorter reaction times for directly related target words presented in the RVF compared to both indirectly related (846ms ± 109 vs 924 ± 160, *p* < .05) and unrelated target words (846ms ± 109 vs 1004 ± 177)  Sceptics’ reaction times to indirectly related targets differed from those to unrelated targets only in the RVF  Believers had shorter reaction times in the LVF for directly related target words compared to unrelated target words (864ms ± 146 vs 1014ms ± 156), and for indirectly related words compared to unrelated target words (912ms ± 177 vs 1014ms ± 156)  Believers had shorter reaction times in the RVF for directly related target words compared to unrelated target words (766ms ± 114 vs 961ms ± 271), and for indirectly related words compared to unrelated target words (875ms ± 199 vs 961ms ± 271).  Believers’ reaction times to directly related targets differed to indirectly related targets only in the RVF (*p* < .005)  **Ns.** difference in reaction times for indirectly related target words presented in the RVF between believers and sceptics (*p* > .03)  **Ns.** difference in believers’ reaction times to directly and indirectly related target words in the LVF  **Ns.** group x VF interaction for directly related or unrelated targets  *Type of priming:*  Group x VF x type of priming interaction (*F*(1, 22) = 10.74, *p* < .005)  For sceptics, direct semantic priming differed from indirect sematic priming in both the LVF (158ms ± 214 vs -23ms ± 92, *p* < .001) and the RVF (158ms ± 112 vs 80ms ± 80, *p* < .05) – shorter reaction times for indirect semantic priming  For believers, direct semantic priming differed from indirect semantic priming in the RVF (196ms ± 180 vs 87ms ± 100, *p* < .001) – shorter reaction times for indirect semantic priming  **Ns.** difference in reaction times between direct and indirect semantic priming in the LVF for believers |
| Irwin & Green (1998-99) | 194 (57.2) | 18-46, 22.2 (6.05) | | CME | Central monitoring computer game | + corr. paranormal belief and cognitive perceptual (*r* = .35, *p* < .001) and disorganised (*r* = .24, *p* < .001) subscales of SPQ-B  **Ns.** corr. paranormal beliefs and interpersonal subscale of SPQ-B  **Ns.** corr. paranormal beliefs and central monitoring performance (*p* > .15) |
| Tobacyk (1983) | 110 (35.5) | *f* /, 20.6 (3.0)  *m* /, 20.1 (1.7) | | CC | RCRG | **Ns.** corr. paranormal beliefs and CC |

*Note: / = information not reported, bl = believers, sc = sceptics, f = females, m = males, ISL = implicit sequence learning, ISP = implicit semantic priming, VF = visual field, LVF = left visual field, RVF = right visual field, CME = central monitoring efficiency, RE = reasoning errors, CC = cognitive complexity, + = positive, - = negative, corr. = correlation,* ***Ns.*** *= nonsignificant, SPQ-B = Schizotypal Personality Questionnaire Brief (Raine & Benishay, 1995), RCRG = Role Construct Repertory Grid (Kelly, 1955)*
