# Supplementary material for: Paranormal beliefs and cognitive function: A systematic review and assessment of study quality across four decades of research
Source: PLoS One. 2022 May 4;17(5):e0267360. doi: 10.1371/journal.pone.0267360 (PMC9067702; doi:10.1371/journal.pone.0267360)
Supplement: S8 Table — Note:† = papers that provided reliability statistics for their novel scales, ‡ = used a translated version of the original scale, * = Musch & Ehrenberg (2002) developed a novel scale that was later named the BPS and was used in two subsequent studies. RPBS = Revised Paranormal Belief Scale (Tobacyk 1988; 2004), ASGS = Australian Sheep-Goat Scale (Thalbourne & Delin, 1993), PBS = Paranormal Belief Scale (Tobacyk & Milford, 1982), Rasch RPBS = Rasch devised Revised Paranormal Belief Scale (Lange et al., 2000), BPS-O = Belief in the Paranormal Scale (Original; Jones et al., 1977), BPS = Belief in the Paranormal Scale (Musch & Ehrenberg, 2002), MMU-N = Manchester Metropolitan University New (see Dagnall et al., 2010), MMU-PS = Manchester Metropolitan University Paranormal Scale (see Dagnall et al., 2010), SSUB = Survery of Scientifically Unsubstantiated Beliefs (Irwin & Marks, 2013), OS = Occultism Scale (Böttinger, 1976), PS = Paranormal Scale (Orenstein, 2002), AEI = Anomalous Experiences Inventory (Gallagher et al., 1994; includes a ‘belief’ subscale). (DOCX) [file pone.0267360.s010.docx]

**S8 Table. Measures of paranormal beliefs used in the 71 studies included in the review.**

| **RPBS** | **Novel** | **ASGS** | **PBS** | **Rasch RPBS** | **Okkulten Glaubens** | **BPS-O** | **BPS*** | **MMU-N** | **MMU-PS** | **SSUB** | **OS** | **PS** | **AEI** |
| --- | --- | --- | --- | --- | --- | --- | --- | --- | --- | --- | --- | --- | --- |
| Morgan & Morgan (1998)  Irwin & Green (1998-99)  Dudley (1999)  Rudski (2004)  Hergovich & Arendasy (2005)  Dagnall et al. (2007)  McLean & Miller (2010)  Lindeman et al. (2011)  Riekki et al. (2013)  Svedholm & Lindeman (2013)  Van Elk (2013)  Willard & Norenzayan (2013)  Blanco et al. (2015)  Van Elk (2015)  Lasikiewicz (2016)  Lindeman & Svedholm-Häkkinen (2016)  Caputo (2017)  Van Elk (2017)  Barberia et al. (2018)‡  Wilson (2018)  Andrews & Tyson (2019)  Mikušková & Cavojavá (2020)  Lesaffre et al. (2020)  Pérez-Navarro & Martínez-Guerra (2020) | Alcock & Otis (1980)†  Brugger et al. (1990)  Brugger et al. (1991)  Blackmore & Moore (1994)  Blackmore (1997)  Bressan (2002)†  Musch & Ehrenberg (2002)†  Lawrence & Peters (2004)  Majima (2015)†  Prike et al. (2017)†  Branković (2019)†  Griffiths et al. (2019)†  Bestch et al. (2020)†  Rizeq et al. (2020) | Roe (1999)  Wilson & French (2006)  Palmer et al. (2007)  Rogers et al. (2009)  Krummenacher at al. (2010)  Dagnall et al. (2014)  Simmonds-Moore (2014)  Gray & Gallo (2016)  Rogers et al. (2016)  Prike et al. (2018) | Tobacyk (1983)  Tobacyk (1984)  Gagné & McKelvie (1990)  Royalty (1995)  Smith et al. (1998)  Greening (2002) | Wain & Spinella (2007)  Dagnall et al. (2014)  Irwin et al. (2014)  Dagnall et al. (2016A)  Dagnall et al. (2016B)  Denovan et al. (2018) | Schienle et al. (1996)  Gianotti et al. (2001)  Pizzagalli et al. (2001) | Wierzbicki (1985)  Roberts & Seager (1999) | Genovese (2005)  Stuart-Hamilton et al. (2006) | Dagnall et al. (2014) | Drinkwater et al. (2019) | Irwin (2015) | Hergovich (2003) | Ståhl & van Prooijen (2018) | Rogers et al. (2019) |
| 24 | 14 | 10 | 6 | 6 | 3 | 2 | 2 | 1 | 1 | 1 | 1 | 1 | 1 |

*Note:† = papers that provided reliability statistics for their novel scales, ‡ = used a translated version of the original scale, * = Musch & Ehrenberg (2002) developed a novel scale that was later named the BPS and was used in two subsequent studies. RPBS = Revised Paranormal Belief Scale (Tobacyk 1988; 2004), ASGS = Australian Sheep-Goat Scale (Thalbourne & Delin, 1993), PBS = Paranormal Belief Scale (Tobacyk & Milford, 1982), Rasch RPBS = Rasch devised Revised Paranormal Belief Scale (Lange et al., 2000), BPS-O = Belief in the Paranormal Scale (Original; Jones et al., 1977), BPS = Belief in the Paranormal Scale (Musch & Ehrenberg, 2002), MMU-N = Manchester Metropolitan University New (see Dagnall et al., 2010), MMU-PS = Manchester Metropolitan University Paranormal Scale (see Dagnall et al., 2010), SSUB = Survery of Scientifically Unsubstantiated Beliefs (Irwin & Marks, 2013), OS = Occultism Scale (Böttinger, 1976), PS = Paranormal Scale (Orenstein, 2002), AEI = Anomalous Experiences Inventory (Gallagher et al., 1994; includes a ‘belief’ subscale)*
