# Supplementary material for: Paranormal beliefs and cognitive function: A systematic review and assessment of study quality across four decades of research
Source: PLoS One. 2022 May 4;17(5):e0267360. doi: 10.1371/journal.pone.0267360 (PMC9067702; doi:10.1371/journal.pone.0267360)
Supplement: S9 Table — Note: ✓ = original category, ✓ = alternate category. (DOCX) [file pone.0267360.s011.docx]

**S9 Table. Alternate categorisations for studies included in the review.**

|  | **Section** | | | | | | |
| --- | --- | --- | --- | --- | --- | --- | --- |
| **Study** | **Perceptual & cognitive biases** | **Reasoning** | **Intelligence, critical thinking & academic performance** | | **Thinking style** | **Executive function & memory** | **Other cognitive functions** |
| Alcock & Otis (1980) | ✓ |  | | **✓** |  |  |  |
| Andrews & Tyson (2018) |  |  | | **✓** |  |  |  |
| Barberia et al. (2018) | **✓** |  | |  |  |  |  |
| Betsch et al. (2020) | ✓ |  | | **✓** | ✓ |  |  |
| Blackmore (1997) |  | **✓** | |  |  | ✓ |  |
| Blackmore & Moore (1994) | **✓** |  | |  |  | ✓ |  |
| Blanco et al. (2015) | **✓** |  | |  |  |  |  |
| Branković (2019) | ✓ |  | |  | **✓** |  |  |
| Bressan (2002) |  | **✓** | |  |  | ✓ |  |
| Brugger et al. (1990) |  | **✓** | |  |  | ✓ |  |
| Brugger et al. (1991) | ✓ | **✓** | |  |  | ✓ |  |
| Caputo (2017) | **✓** |  | |  |  |  |  |
| Dagnall et al. (2007) |  | **✓** | |  |  | ✓ |  |
| Dagnall et al. (2014) |  | **✓** | |  |  | ✓ |  |
| Dagnall et al. (2016A) |  | **✓** | |  |  | ✓ |  |
| Dagnall et al. (2016B) |  | **✓** | |  |  | ✓ |  |
| Denovan et al. (2018) |  | **✓** | |  |  | ✓ |  |
| Drinkwater et al. (2019) | **✓** |  | |  |  |  |  |
| Dudley (1999) |  |  | |  |  | **✓** |  |
| Gagné & McKelvie (1990) | **✓** | ✓ | |  |  | ✓ |  |
| Genovese (2005) |  |  | |  | **✓** |  |  |
| Gianotti et al. (2001) | ✓ |  | |  | **✓** |  |  |
| Gray & Gallo (2016) |  |  | | ✓ | ✓ | **✓** |  |
| Greening (2002) |  |  | |  |  | **✓** |  |
| Griffiths et al. (2019) | **✓** | ✓ | |  |  | ✓ |  |
| Hergovich (2003) | ✓ |  | |  | **✓** | ✓ |  |
| Hergovich & Arendasy (2005) |  | ✓ | | **✓** |  | ✓ |  |
| Irwin (2015) |  |  | |  | **✓** |  |  |
| Irwin & Green (1998-99) |  |  | |  |  | ✓ | **✓** |
| Irwin et al. (2014) | **✓** | ✓ | |  |  | ✓ |  |
| Krummenacher et al (2010) | **✓** |  | |  |  | ✓ |  |
| Lasikiewicz (2016) |  |  | |  | **✓** |  |  |
| Lawrence & Peters (2004) |  | **✓** | |  |  | ✓ |  |
| Lesaffre et al. (2020) | **✓** | ✓ | |  |  |  |  |
| Lindeman & Svedholm-Häkkinen (2016) |  |  | |  | **✓** | ✓ |  |
| Lindeman et al. (2011) |  |  | |  |  | **✓** |  |
| Majima (2015) |  |  | | ✓ | **✓** |  |  |
| McLean & Miller (2010) |  |  | | **✓** |  |  |  |
| Mikušková & Cavojavá (2020) | ✓ | ✓ | |  | **✓** |  |  |
| Morgan & Morgan (1998) |  |  | | **✓** |  |  |  |
| Musch & Ehrenberg (2002) |  | **✓** | | ✓ |  | ✓ |  |
| Palmer et al. (2007) |  | ✓ | |  |  | ✓ | **✓** |
| Pérez-Navarro & Martínez-Guerra (2020) |  | **✓** | |  |  | ✓ |  |
| Pizzagalli et al. (2001) |  |  | |  |  |  | **✓** |
| Prike et al. (2017) |  | **✓** | |  |  | ✓ |  |
| Prike et al. (2018) | **✓** | ✓ | |  |  | ✓ |  |
| Riekki et al. (2013) | **✓** |  | |  |  | ✓ |  |
| Rizeq et al. (2020) |  |  | | ✓ | **✓** | ✓ |  |
| Roberts & Seager (1999) |  | **✓** | |  |  | ✓ |  |
| Roe (1999) |  |  | | **✓** |  |  |  |
| Rogers et al. (2009) |  | **✓** | |  |  | ✓ |  |
| Rogers et al. (2016) | ✓ | **✓** | |  |  | ✓ |  |
| Rogers et al. (2019) |  |  | |  | **✓** |  |  |
| Royalty (1995) |  | ✓ | | **✓** |  | ✓ |  |
| Rudski (2004) | **✓** | ✓ | |  |  | ✓ |  |
| Schienle et al. (1996) | **✓** | ✓ | |  |  | ✓ |  |
| Simmonds-Moore (2014) | **✓** |  | |  |  | ✓ |  |
| Smith et al. (1998) |  |  | | **✓** |  | ✓ |  |
| Ståhl & van Prooijen (2018) |  |  | | ✓ | **✓** |  |  |
| Stuart-Hamilton et al. (2006) |  | ✓ | | **✓** |  | ✓ |  |
| Svedholm & Lindeman (2013) |  |  | |  | **✓** | ✓ |  |
| Tobacyk (1983) |  | ✓ | |  |  | ✓ | **✓** |
| Tobacyk (1984) |  |  | | **✓** |  | ✓ |  |
| Van Elk (2013) | **✓** |  | |  |  | ✓ |  |
| Van Elk (2015) | **✓** |  | |  |  | ✓ |  |
| Van Elk (2017) | **✓** | ✓ | |  |  | ✓ |  |
| Wain & Spinella (2007) |  |  | |  |  | **✓** |  |
| Wierzbicki (1985) |  | **✓** | |  |  | ✓ |  |
| Willard & Norenzayan (2013) | **✓** |  | |  |  |  |  |
| Wilson (2018) |  |  | | **✓** |  |  |  |
| Wilson & French (2006) |  |  | |  |  | **✓** |  |

*Note:* **✓** *= original category,* ✓ *= alternate category*
