# Supplementary material for: Paranormal beliefs and cognitive function: A systematic review and assessment of study quality across four decades of research
Source: PLoS One. 2022 May 4;17(5):e0267360. doi: 10.1371/journal.pone.0267360 (PMC9067702; doi:10.1371/journal.pone.0267360)
Supplement: Attachment — Submitted filename: Response to Reviewers.docx [file pone.0267360.s012.docx]

**Manuscript Submission ID PONE-D-21-32750**

**Response to Reviewers**

Dear Dr Perales,

Thank you for giving us the opportunity to submit a revised version of the manuscript “Paranormal beliefs and cognitive function: A systematic review and assessment of study quality across four decades of research” for publication in PLOS ONE. We appreciate the time and effort that you and the reviewers committed to providing feedback on our manuscript, and we are grateful for the insightful and detailed comments. Please see below for a point-by-point response to the reviewers’ comments, with our responses written in blue. All page and line numbers refer to the revised manuscript file with changes highlighted in blue.

We would like to thank you and the reviewers again for the valuable comments, which we feel have greatly improved the manuscript. We look forward to hearing from you.

Sincerely,

Charlotte E. Dean

PhD Candidate

Department of Psychology, Sport and Geography

School of Life and Medical Sciences

University of Hertfordshire

College Lane

Hatfield

Herts AL10 9AB

United Kingdom

**Reviewers’ Comments to the Authors:**

**Reviewer 1**

The authors present a review article on the association between paranormal beliefs and divergencies in cognitive function. The topic has been gaining relevance in the last decades in the research community and is of interest for a wide audience. Although I agree with the authors that a metanalysis would have been more valuable, I reckon that the heterogeneity in the studies published so far hinders that approach. In any case, I believe that a systematic review paper like the one presented will be of use for many researchers interested in this field during the following years.

**Author response:** We would like to thank the reviewer for the detailed feedback provided below.

1. Line 87: I think there is a parenthesis missing (or one too many)

**Author response:** This section has been moved and a parenthesis removed (the revised text can be found on page 14 of the revised manuscript, line number 290-293).

2. Line 95: I guess that this is a matter of personal preference (so you can ignore it), but I thought the sentences on line 95 to 100 (“While factors…”) were not very relevant for the study (which is already quite long and includes many references) so they could be deleted.

**Author response:** This section of text has been removed.

3. Line 101: While setting the case for the study of cognitive function and paranormal beliefs, the authors comment on studies of “other” kinds of beliefs, such as religious beliefs and conspiracist beliefs. Regarding the former, as far as I know, religious beliefs are, at least sometimes, considered one type of paranormal beliefs (see the Traditional Religious Beliefs dimension in the widely used questionnaire by Tobacyk). I think this should be clarified in the text. As for conspiracist beliefs, the authors could make use of the concept of “epistemically unwarranted beliefs” (see Lobato et al., 2014) to stablish links between these types of beliefs and maybe include some references to pseudoscientific beliefs too.

**Author response:** While religious beliefs are sometimes considered to be a component of paranormal beliefs, there is contradictory evidence concerning the relationship between these two belief clusters. In the context of the cited studies (Hosseini et al., 2019; Kaufman et al., 2007), religious and spiritual beliefs were identified and treated as their own belief types separate from belief in paranormal phenomena. While we acknowledge that there are many belief types that could be included here, this is only intended to offer a brief example of the relationships between cognition and other belief clusters. However, we have referenced Lobato et al.’s (2014) work and the concept of “epistemically unwarranted beliefs”. The revised text can be found on page 4 of the revised manuscript (line number 90-91).

4. Line 114: Irwin’s review only included null findings ?

**Author response:** This section has been rewritten to include a quote from Irwin’s (1993) paper for clarity. The revised text can be found on page 5 of the revised manuscript (line number 99-102). The revised text reads as follows:

“That review, however, was published almost 30 years ago, was not systematic, and may have introduced some bias “…citing null results only when these form a substantial proportion of the available data on a given relationship” (p.6).”

5. Line 133: The aims of the larger study should be (at least briefly) explained.

**Author response:** The aims of the larger study (to also assess the relationships between paranormal beliefs and schizotypal personality traits) have briefly been explained, and the revised text can be found on page 5 of the revised manuscript (line number 113-114).

6. Line 143: Were any unpublished works or theses included in the final list of studies. If so, this should be clarified through the description of the process and, at least, when discussing the final selection.

**Author response:** One unpublished doctoral thesis was included in the final list of studies, and this has been highlighted at the start of the results section when discussing the final selection (page 8 of the revised manuscript, line number 164-165).

7. (around) Line 177: The “sought for retrieval” step appears in Figure 1, but I think it is not explained in the text.

**Author response:** The text has been amended to clarify that full-text copies were unavailable for five studies which were subsequently sought for retrieval (page 6 of the revised manuscript, line number 134-136).

8. Line 207: I do not think illusion of control and causal illusion should be categorized as perceptual biases. As far as I know, they are usually characterized as cognitive biases (see Matute, Yarritu and Vadillo, 2011), and they are very different to the other studies included in this section (visual noise studies).

**Author response:** These studies have been moved to the summary of studies concerning cognitive biases. The revised text can be found on page 19 of the revised manuscript (line number 418-420).

9. Line 212: Here, I missed inclusion of Torres et al. (2020) (it appeared in a Scopus search using “paranormal belief” AND cogni*)

**Author response:** Torres et al. (2020) was picked up during the Scopus search and was one of the 902 initial papers found from the electronic and hand searches. While the abstract mentions paranormal phenomena, pseudoscientific beliefs were the primary focus of the study (e.g., beliefs about health conditions). This study was therefore excluded from the final selection of papers as it presented original data relating to a measure of pseudoscientific beliefs, rather than a measure of paranormal beliefs.

10. Lines 221 and 227: In which unit are these quantities expressed and how do they relate with the percentages in line 217

**Author response:** These quantities have been removed to improve the clarity of the text here. The revised text can be found on page 18 of the revised manuscript (line number 382-386).

11. 205: How were paranormal beliefs measured in each study?

**Author response:** A discussion of the paranormal belief measures used in each study has been included in the general discussion. The revised text can be found on page 33 of the revised manuscript (line number 748-756). A new supplementary table (S8 Table) has also been provided which identifies the paranormal belief measure used in each study.

12. Lines 287 and 295: Aren’t repetition avoidance tasks a measure of probabilistic reasoning? I think these studies would fit more comfortably in the next section.

**Author response:** The text here has been amended and one of these studies has been moved to the “Reasoning” subsection (see page 23 of the revised manuscript; line number 511-514).

13. Line 370: associations between paranormal beliefs and what?

**Author response:** The text has been amended and rearranged here to improve clarity. The revised text can be found on pages 25-26 of the revised manuscript.

14. Line 372: Here, I missed Barberia et al. (2018) (also appeared in a Scopus search).

**Author response:** We acknowledge the reviewer’s comment and have added this study to the review (page 20 of the revised manuscript; line number 444-446). Statistical tests, figures and tables presented with the manuscript have been revised to reflect this change.

15. Line 378: reference 125 is about an association between pseudoscientific beliefs and paranormal beliefs. I am not sure this should be included as a (cognitive) measure of critical thinking given the relation between the two types of beliefs. If the authors decide to keep it, then they should also consider other studies relating different types of unwarranted beliefs with each other (e.g., Lobato et al, 2014; Fasce and Pico, 2019…).

**Author response:** We acknowledge and agree with the reviewer’s comment and have removed this study from the review. Statistical tests, figures and tables presented with the manuscript have been revised to reflect this change.

16. Line 439: In this section I missed Meyersburg et al. (2009) study, but then I couldn’t find it when I tried a search in Scopus so I guess it is ok.

**Author response:** Unfortunately, this study was not picked up through our electronic or hand searches.

17. Line 507: when discussing sampling representativeness, I think the sex/gender issue deserves some comment. Were samples composed of both men and women? were they balanced? At least in those studies recruiting Psychology students my guess is that more women would have been involved…

**Author response:** A discussion of sex/gender issues relating to sample representativeness has been added to the text and can be found on pages 13-14 of the revised manuscript. We note here that of all samples, “most participants are also female (>60%)” (line number 273). This has been discussed in relation to the (inconsistent) reporting of gender effects for paranormal belief.

18. Line 528: I would appreciate a table or at least more information on the percentages of usage of each different test (not just Tobacyk and ad hoc questionnaires). It could be even worth providing information separated by each topic . Could differences regarding the association between critical thinking and paranormal beliefs be related to the use of different measures of belief endorsement?

**Author response:** As mentioned above, details of this have been included in the discussion detailing information relating to the paranormal belief measures used in each study (line number 748-756), and a new supplementary table (S8 Table) has been provided. We have also acknowledged within the text that “this variation most likely contributes to heterogeneity across studies and potentially undermines the reliability of reported associations between cognitive functions and paranormal beliefs.” (line number 753-755).

19. Line 683: I think the authors should include in the discussion some comment on the implications of the fact that most of the studies analyzed are correlational.

**Author response:** We have commented on the implications of the largely correlational approach of the studies included in the review. The revised text can be found on page 33 of the revised manuscript (line number 775-779).

20. Line 755: Regarding the proposal of the fluid-executive theory, the authors should describe ways in which this hypothesis could be tested.

**Author response:** We have briefly described how this could be tested in our revised discussion of the fluid-executive model, using between-subjects designs matching groups of believers and sceptics for fluid intelligence. The revised text can be found on page 37 of the revised manuscript (line number 856-857).

**Reviewer 2**

The authors present a systematic review (without meta-analysis) of the literature relating paranormal beliefs with performance in different cognitive tasks, with a particular focus in the critical assessment of the quality of the studies conducted so far. Overall, I think that this can be a valuable resource for researchers working on this area and, hopefully, it will also contribute to improving the quality of future research. I do have some concerns, though, that the authors might want to address in the final version of the manuscript.

**Author response:** Thank you for the detailed comments and careful review.

1. Perhaps my most important concern is that in the present version of the ms it is quite difficult to follow the results section. As the authors themselves acknowledge their classification of tasks is somewhat arbitrary because “such classifications are necessarily a simplification and are not intended to be a definitive organisation”. This is undeniable true, but even so some of the headings collate results from radically different tasks and at some points I couldn’t help thinking that some paragraphs actually belonged in a different section. Or, perhaps alternatively, the authors might want to provide some explanation for the logic behind including several tasks under a common heading. This is particularly problematic in the section now titled “Cognitive and perceptual biases”, which includes a wide range of phenomena from illusion of control (measured in learning tasks comprising hundreds of trials) to perception of faces and other stimuli. What’s the common feature underlying these different tasks? Is it the case that all (or most) of them are somehow related to the (illusory) perception of patterns where there is just noise? If that’s the case, I think the authors need to spell this logic out more clearly, possibly change the name of the section and perhaps move some of the tasks that do not fit well in this category to other sections. They might even want to consider dividing this section in two or more less heterogeneous sections. I also found it slightly odd that the jump to conclusions task is included in this section, when it is actually more similar to some of the probabilistic reasoning tasks included in the following section.

**Author response:** We have tried to make the somewhat arbitrary categorisation of studies clearer throughout the results section. In addition to revising the text to group studies together more clearly, we have included a new supplementary table (S9 Table) as an effort to more clearly show how each study could contribute to multiple categories. Unfortunately, the high variability in outcome measures within studies hinders a more definitive organisation.

2. Also, in the present version it is easy to get lost in the enumeration of Results, without getting a glimpse at the whole picture until the Discussion section. If the results section were briefer this would be ok, but given the length of the paper I think many readers would find it useful to be reminded occasionally of the interim conclusions that can be reached with the information provided in each subheading. In other words, I miss 1-2 concluding sentences providing an overview of the results found for each category of tasks.

**Author response:** We agree that, given the length of the paper, interim conclusions for each subsection of the results section would be beneficial. We have now included short summaries following each subsection to provide a clearer overview of the results.

3. The assessment of the quality of the studies plays a very important role in the manuscript and it is indeed a great contribution. But while reading it I had some concerns about the reduction of this information to a single “quality score” for each study. Although this is certainly common in meta-analytic literature, this approach has also been criticized, and rightly so in my opinion (e.g., Jüni, P., Witschi, A., Bloch, R., & Egger, M. (1999). The hazards of scoring the quality of clinical trials for meta-analysis. JAMA, 282, 1054–1060.) I would not ask the authors to change their approach, but I think it would be worth mentioning, even if briefly, that reducing the responses to these quality scales to a single score can be misleading and should be taken with caution.

**Author response:** We agree with the concerns about the reduction of study quality to a single metric and have added this to the discussion of limitations relating to the present work. The revised text can be found on page 37 of the revised manuscript (line number 863-867).

4. In the introduction, the authors mention that the two most common tests to measure paranormal beliefs have good psychometric properties. But this is just one side of the story. These studies are trying to relate paranormal beliefs with performance in cognitive tasks that might not have such good psychometric properties. See, e.g., Hedge, C., Powell, G., & Sumner, P. (2018). The reliability paradox: Why robust cognitive tasks do not produce reliable individual dif- ferences. Behavior Research Methods, 50, 1166–1186. https://doi. org/10.3758/s13428-017-0935-1 This is important, because the observed correlation between any two measures is attenuated downwards if any of them (not just the paranormal belief scale) is unreliable. If the reliability of these tasks does not improve in future research (or sample sizes are not adjusted taking it into account) this will necessarily result in high heterogeneity (i.e., much more variance from one study to another) and small average effect sizes.

**Author response:** This is an interesting and important issue, and a discussion of this can be found on pages 33-34 (line number 757-773) of the revised manuscript.

5. Regarding sample sizes, I was a bit puzzled to read that “overpower” can be a problem in this area. I am afraid I strongly disagree with this point of view. In my opinion, there cannot be such a thing as an excess of power. The authors justify this saying that “… large studies might also be over-powered and thus, detecting very small and possibly trivial effects”. This confounds hypothesis testing (whether an effect is significantly different from zero) with parameter estimation (what the exact size of an effect). The fact that many researchers take a significant result for a “relevant” result is the consequence of using statistical tests mindlessly and it would be an error to encourage researchers to use smaller-than-possible sample sizes as a solution. Regardless of whether you want to test the null hypothesis or know the exact size of an effect, a large sample size will always be helpful because it will reduce the uncertainty of your inferences. What researchers need to be reminded is that not everything that is statistically significant is important.

**Author response:** We acknowledge the points raised by the reviewer and have deleted the discussion of overpowering from the manuscript.

6. If the goal is to represent a trend in time, I suspect that most readers would find Figure 5 easier to read if years are plotted in the x-axis.

**Author response:** Figure 5 has been amended so that “Years” are plotted in the x-axis.

7. In the flow chart, I couldn’t understand how from 475 records screened you take 5 reports for retrieval, but then below you have again 84 reports originating from these 5. Something seems to be wrong or misleading in the flow of records.

**Author response:** The text has been amended to clarify that full-text copies were unavailable for five studies which were subsequently sought for retrieval (page 6 of the revised manuscript, line number 135-136).

**Reviewer 3**

This paper provides a comprehensive and thorough review of research into the relationship between paranormal beliefs and cognitive functioning. As the authors note, considerable time has passed since the last review of paranormal beliefs and cognition, so this paper makes a strong, important, and timely (if not long overdue) contribution to the literature. I also commend the authors for preregistering their PRISMA guidelines and for the level of detail and clarity they provide about how the systematic review was conducted. Overall, I think this paper will make a great contribution to the literature. However, there are some areas where I believe the paper could be improved which I have highlighted in detail below.

**Author response:** Thank you for the comments and insightful review.

1. Regarding the paper structure, I think it might be better to more clearly separate the study quality assessment results and discussion from the paranormal belief and cognition findings and theory results and discussion. The Introduction focuses on the key points, is clear and easy to follow, and provides an appropriate (broad) set up for the focus of the paper. Similarly, the Method section is clear, concise, and enjoyable to read. However, from the Results onwards the paper can be quite difficult to follow in places. For example, it goes from the Method to an outline of findings in the literature, then to sections on study quality assessment, then back to a discussion of cognitive deficits/differences, then onto open science (I think this section should be moved to be alongside the sampling issues and non-respondents), then back onto a summary of research findings and a proposal for a new theory etc. This review paper is doing two things. Firstly, extracting and assessing the quality of the existing literature on paranormal beliefs and cognitive function. Secondly, it is also outlining and synthesising the findings from that literature (plus proposing a new theory/hypothesis for testing). These are two separate and quite distinct focuses. Therefore, I think it would be better to more clearly delineate them and instead present all of one aspect (quality assessment and relevant discussion) followed by everything relevant to the other (outline and synthesis of findings).

**Author response:** The manuscript has been rearranged so that the assessment of study quality is presented prior to the outline and synthesis of findings from the narrative review. The assessment of study quality and related issues can be found on pages 8-16 of the revised manuscript.

2. Related to the above point, the results generally provide a clear and comprehensive summary of the various findings that have been catalogued within each subsection. However, I think each subsection would benefit from an overall summary or synthesis that brings it all together. If you make the changes to the paper structure that I have recommended above, this may no longer be necessary because these results will be more closely followed by a relevant discussion section (but see how it looks and consider it). In comparison, the results for the quality assessment are accompanied by relevant discussion within the actual results section.

**Author response:** As noted above, we agree that the addition of subsection summaries would improve the overall clarity of the results, and short summaries following each subsection have now been included in the revised manuscript.

3. I think the proposed fluid-executive theory is underdeveloped and would benefit from some further explanation and elaboration. You explain how it would relate to probabilistic reasoning but don’t outline how it would contribute to or explain the other findings (e.g., cognitive and perceptual biases). Additionally, I think you need to do more work to justify why this specific aspect should be focused on, rather than alternative explanations. For example, others might argue that analytical thinking (Pennycook et al., 2015), or a “rationality quotient” (Stanovich, 2016; Stanovich et al., 2016; Weller, 2017; although see Ritchie, 2017), could also be proposed as underlying (or overarching) theories that explain the various associations between paranormal belief and cognitive functioning. I am happy to be convinced that the proposed theory is the best/most plausible candidate, I just think it needs some further fleshing out and additional evidence to support it.

**Author response:** We agree that the discussion of the proposed theory/model required additional detail. The revised text can be found on pages 34-37 of the revised manuscript (under the new heading of “Future directions – the fluid-executive model”; line number 780-857).

4. I understand the desire to not include studies on children, given the potential cognitive differences. However, from examining Table S1, it seems that for all the excluded studies the vast majority of participants included in the studies were adults and they just happened to also include some teenagers in the study. I think it would be justifiable to exclude studies that had solely focused on children or teenage samples but given the already wide variability in cognitive function between 18- and 70-year olds, it seems unnecessary to exclude these studies solely because they also include some participants in their mid-late teens. I know this is a deviation from your preregistration so feel free to push back or disagree, but it wouldn’t change your conclusions and I think it’s okay to make some deviations if they are well justified.

**Author response:** As the preregistered protocol contained specific inclusion/exclusion criteria, we have not included these studies in the final (revised) review. While the inclusion or exclusion of these studies does not change the overall conclusions of the review, we do acknowledge that this is a potential limitation of the current work. As such, we have discussed this on pages 37-38 of the revised manuscript (line number 868-877).

5. Is the discussion of test theories and differential item functioning in the Introduction necessary? It seems like an unnecessary distraction from the main focus of the paper, so I’d just leave it at a sentence or two explaining that there is debate about why these differences are found.

**Author response:** This discussion has been moved to the discussion of study quality, where it provides more relevant support to the discussion of sample representativeness and a bias towards female participants in the included studies.

6. I think that Figures 2-4 for the AXIS data would be greatly improved if you also included No and Unsure (you could keep the current format but have the bars contain different colours for each response category). This is particularly important because when initially looking at the figures it is not clear that there was an “unsure” category (e.g., it looks like half the studies didn’t have ethical approval, when it likely just wasn’t explicitly reported).

**Author response:** We agree that these figures could be more clearly presented, and we have made the suggested changes to Figures 2-4.

7. The section on open science focuses solely on pre-registration but there are many other aspects of open science such as publicly sharing data, analysis scripts, materials etc. I think you should either broaden this section to cover those additional open science aspects or, if you want to avoid lengthening the paper, then you could combine the pre-registration section with the sample size justification section, presenting it as a possible solution to address these problems.

**Author response:** The discussion of pre-registration has been combined with the discussion of sample size justification. The revised text can be found on page 11-12 of the revised manuscript, under the revised heading “Sample size justification, sample representativeness and open science” (line number 228-248).

8. The data are not currently accessible via the OSF link (https://osf.io/7bthg/). Please update the OSF page to make it public or provide a reviewer only link if you don’t want to make the data fully open to the public yet. Don’t worry, I’ve done the same thing before and this happens with half or more of the OSF links I’ve seen when reviewing papers.

**Author response:** Please find below a reviewer-only link to the datafiles on the OSF page. We would like to assure the reviewers that the data will be made publicly available upon publication of the manuscript.

Reviewer-only OSF link:

<https://osf.io/7bthg/?view_only=4761e1c07ad249ebacd8f88f660b072b>

**Reviewer 4**

The paper presents a review and summary of the past 40 years of research on paranormal belief and cognitive functioning. As noted by the authors, there has not been a systematic review of this relationship since Irwin’s (1993) work almost 30 years ago. I wholeheartedly agree with the authors that such a systematic review is needed, and that it would add significantly to our overall understanding of the current state of the field. Unfortunately, though, I think there are some key issues with the current attempt that need to be addressed to turn it into a beneficial contribution to the area.

**Author response:** We would like to thank the reviewer for their comments and detailed review.

1. Given the range of tasks and variables in this particular area, I have no doubt it was difficult to synthesize the information in a straightforward and simple manner. However, even though I work in this area, I found it hard to track through the main sections. Specifically, each section was a listing of how one study showed X, two studies showed Y, et cetera, and by the end of each section it was not clear what specifically the reader should take away. At a minimum, using something like clear and specific tables to help organize the material would help immensely, especially in terms of trying to track through what the various studies do or do not show. There are the tables in the supplementary material, and admittedly even though they are broken up by section and a bit tricky to see “overall” outcomes, I found them easier to follow in terms of thinking across the studies. Relatedly, in several spots the writing/presentation was dense, which may have added to the experience of not knowing what the “take home” message was for each section. For example, proper paragraphs should rarely run over a page, but more than one did, and one paragraph actually went for almost a full 2 pages (pgs 11-13). In general, editing for direct language, paragraph length, et cetera, would help improve clarity of the information being presented.

**Author response:** As noted above, we have amended the text throughout the results section to increase the clarity of the manuscript. To support the identification of “overall” outcomes, a summary has been provided for each subsection of the results. We have also included an additional supplementary table (S9 Table) which highlights that many of the studies could make contributions to several categories.

2. Again, I understand it would be difficult to categorize the experiments, but the current way of doing it seems to actually work against providing a systematic review. For example, there were fewer studies than I would have expected in the thinking styles section, just because this has been a particularly popular topic to explore in terms of paranormal beliefs. I could see, though, how some of that work would have ended up in other sections given the classification criteria; however, it then feels like we are not getting the full picture. Again, I think this is where tables may be particularly useful; for example, rather than binning studies under just one section, it would be much more useful to have tables that include all of the categories. Thus, we would be able to see what each study contributes across the categories (when relevant), rather than to just a single category. I recognize the authors may have attempted this approach and for some reason it was not viable, but based on systematic reviews in other areas that have used this set-up it would seem to be the more useful approach. This type of set-up would also help more clearly and succinctly demonstrate what the reader should take away from the area.

**Author response:** Again, we have created a new supplementary table (S9 Table) containing all of the categories as an effort to more clearly show where studies could contribute to multiple categories.

3. The limitations discussion seems like a bit of a tacked-on section rather than a real consideration of the current work. For example, as already mentioned, one potential issue is how the studies had to be categorized, which means they can only contribute to one section even though they may potentially also be able to contribute to at least one other section.

**Author response:** The limitations section has been rewritten to provide a more detailed consideration of the current work. The revised text can be found on pages 37-38 of the revised manuscript.

4. Further, and this may be an unfair criticism given the complexity of the area, but I had fully expected at least some sort of meta-analysis of the studies. Again, this may be because the listing out of the studies across the sections did not land well in terms of what to concretely take away. However, given the current techniques available for meta-analyses it feels like that is what we would gain the most from in terms of understanding the current state of the relationships between paranormal belief and cognitive functioning.

**Author response:** We agree that meta-analyses provide a valuable contribution to any literature review and identify this in the manuscript as a limitation of the current work (line number 859-862). Unfortunately, the high level of heterogeneity identified throughout the review (stemming, in part, from the range of both cognitive and paranormal belief measures) and the variability in outcome measures meant that the available data were not conducive to a meta-analytic assessment.

5. A minor point, but was any effort put into searching for articles using the term “anomalistic”? The term “paranormal” is still the most common terminology, but given Chris French and colleague’s focus on using the more broad term of anomalistic belief (and subsequent work from others that has followed suit), it is not clear whether just using “paranormal” would have picked up all of the relevant studies.

**Author response:** The term “anomalistic” was not included as a search term for the electronic database searches as this term is used to describe different phenomena and belief types. We acknowledge the use of “anomalistic belief” as a broader term used within the literature to refer to a range of unconventional beliefs (including paranormal beliefs) but note that the terms “anomalistic” and “paranormal” are often used in conjunction with one another. Indeed, several of the included studies make use of both terms within their text (e.g., Rogers et al., 2019; Prike et al., 2018, 2017; Dagnall et al., 2016A, 2016B). While it is possible that searches using this term may have picked up a few additional papers, the use of the commonly used term “paranormal” within our searches did also return papers using the term “anomalistic”.

References:

Dagnall, N., Denovan, A., Drinkwater, K., Parker, A., & Clough, P. (2016A). Toward a better	understanding of the relationship between belief in the paranormal and statistical bias:	The potential role of schizotypy. *Frontiers in Psychology, 7*, 1045.

Dagnall, N., Drinkwater, K., Denovan, A., Parker, A., & Rowley, K. (2016B). Misperception	of chance, conjunction, framing effects and belief in the paranormal: A further	evaluation. *Applied Cognitive Psychology, 30,* 409-419.

Hosseini, S., Chaurasia, A., & Oremus, M. (2019). The effect of religion and spirituality on	cognitive function: A systematic review. *The Gerontologist, 59*(2), e76-85.

Kaufman, Y., Anaki, D., Binns, M., & Freedman, M. (2007). Cognitive decline in Alzheimer	disease. Impact of spirituality, religiosity, and QOL. *Neurology, 68*(18), 1509-1514.

Lobato, E., Mendoza, J., Sims, V., & Chin, M. Examining the relationship between	conspiracy theories, paranormal beliefs, and pseudoscience acceptance among a	university population. *Applied Cognitive Psychology, 28*(5), 617-625.

Prike, T., Arnold, M.M., & Williamson, P. (2017). Psychics, aliens, or experience? Using the	Anomalistic Belief Scale to examine the relationship between belief and probabilistic	reasoning. *Consciousness and Cognition, 53,* 151-164.

Prike, T., Arnold, M.M., & Williamson, P. (2018). The relationship between anomalistic	belief and biases of evidence integration and jumping to conclusions. *Acta	Psychologica, 190*, 217-227.

Rogers, P., Hattersley, M., & French, C.C. (2019). Gender role orientation, thinking style	preference and facets of adult paranormality: A mediation analysis. *Consciousness	and Cognition, 76,* 102821.
