# Supplementary material for: Paranormal beliefs and cognitive function: A systematic review and assessment of study quality across four decades of research
Source: PLoS One. 2022 May 4;17(5):e0267360. doi: 10.1371/journal.pone.0267360 (PMC9067702; doi:10.1371/journal.pone.0267360)
Supplement: Attachment — Submitted filename: Response to Reviewers (2).docx [file pone.0267360.s013.docx]

**Manuscript Submission ID PONE-D-21-32750R1**

**Response to Reviewers**

Dear Dr Perales,

Thank you for giving us the opportunity to re-revise our manuscript “Paranormal beliefs and cognitive function: A systematic review and assessment of study quality across four decades of research” and for considering its publication in PLOS ONE. We appreciate the time and effort that you and the reviewers committed to providing feedback on both versions of our manuscript (original and first revision), and the detailed comments provided by each of you which we feel have greatly benefitted the work. Please see below for a point-by-point response to the reviewers’ most recent comments, with our responses written in blue. All page and line numbers refer to the re-revised manuscript file with changes highlighted in blue.

We would like to thank you and the reviewers again for the valuable comments, and for the chance to submit a re-revised version of the manuscript for publication. We look forward to hearing from you.

Sincerely,

Charlotte E. Dean

PhD Candidate

Department of Psychology, Sport and Geography

School of Life and Medical Sciences

University of Hertfordshire

College Lane

Hatfield

Herts AL10 9AB

United Kingdom

**Editor’s Comments to the Authors:**

1. Please review your reference list to ensure that it is complete and correct. If you have cited papers that have been retracted, please include the rationale for doing so in the manuscript text, or remove these references and replace them with relevant current references.

**Author response:** Having reviewed the reference list, reference 57 (Bracket & Batten, 2020) has been removed and replaced with a relevant current reference (Mallet et al., 2012) that is publicly published and available. This new reference is identified as reference 57 in the revised manuscript. Some duplicate references were identified within the reference list which have been removed and the reference numbers subsequently updated.

**Reviewers’ Comments to the Authors:**

**Reviewer 1**

1. PRISMA for abstracts:
   a. The Methods section should specify the inclusion and exclusion criteria for the review, and the dates when each database was last searched. It should also specify the methods used to assess risk of bias in the included studies.

**Author response:** The method section of the abstract has been amended to more clearly reflect the information highlighted in the reviewer’s comments. The revised text can be found on page 2 of the revised manuscript (line numbers: 24-31) and reads as follows:

“We searched four databases (Scopus, ScienceDirect, SpringerLink, and OpenGrey) from inception until May 2021. Inclusion criteria comprised papers published in English that contained original data assessing paranormal beliefs and cognitive function in healthy adult samples. Study quality and risk of bias was assessed using the Appraisal tool for Cross-Sectional Studies (AXIS) and results were synthesised through narrative review. The review adhered to the Preferred Reporting Items for Systematic Reviews and Meta-Analyses (PRISMA) guidelines and was preregistered as part of a larger registration on the Open Science Framework (https://osf.io/uzm5v).”

b. The Results section should indicate the number of included studies and participants for each relevant outcome mentioned (e.g., association between paranormal belief and intuitive thinking bias).

**Author response:** The results section of the abstract has been amended to include both the number of included studies and the total number of participants for each of the cognitive domains. The total number of participants has also been included in each section in the main body of text. The revised text for the results section of the abstract can be found on page 2 of the revised manuscript (line number: 32-36) and reads as follows:

“From 475 identified studies, 71 (n=20,993) met our inclusion criteria. Studies were subsequently divided into the following six categories: perceptual and cognitive biases (k=19, n=3,397), reasoning (k=17, n=9,661), intelligence, critical thinking, and academic ability (k=12, n=2,657), thinking style (k=13, n=4,100), executive function and memory (k=6, n=810), and other cognitive functions (k=4, n=368).”

c. The primary source of funding for the review should be specified

**Author response:** No external funding was received for the review. An additional line has been added to the end of the abstract to reflect this, which can be found on page 3 of the revised manuscript (line number 50).

2. PRISMA checklist (manuscript)
   a. Item 5: the Methods section should specify how studies were grouped for the synthesis.
   b. Item 10: the Methods section should specify whether all results that were compatible with each outcome domain in each study were sought (e.g. for all measures, time points, analyses), and if not, the methods used to decide which results to collect.
   c. Item 11/12/14: If I understand it correctly, description of methods for assessment of risk of bias should be presented in the Methods section. Now they are described in the Results section.
   d. Item 13/15: I could not identify descriptions corresponding to these items in the Methods section.

**Author response:** While the description of methods for assessment of quality and risk of bias has been presented in the results section, we feel that describing this alongside the results of the assessment (and following the description of study selection) helps the readability of the manuscript. The PRISMA guidelines require that this information is provided and suggest that this would be found in the method section. We have ensured that information relevant to items 11, 12 and 14 of the PRISMA checklist have been included in the review but have presented these where we felt would be most beneficial to the reader. Comments from the previous round of revision also suggested that the quality and bias assessment would be best placed here.

The “data extraction” subsection of the method has been rewritten to provide a more detailed description of PRISMA 5, 10, 13, and 15. The revised text can be found on pages 8-9 of the revised manuscript (line number: 157-174) and reads as follows:

“We categorised eligible outcome measures broadly to include both global cognitive function and domain-specific cognitive functions. Any measure of cognitive function was eligible for inclusion (e.g., neuropsychological tests, self-report measures). Results for both paranormal beliefs and cognitive functioning could be reported as an overall test score that provides a composite measure, subscale scores that provide domain-specific measures, or a combination of the two. When multiple cognitive outcomes were investigated, we included all measures. To assess the strength of the relationships between paranormal beliefs and various cognitive functions, we calculated the number of positive, negative, or null findings reported by each study included in the review. Measures of paranormal belief were examined to determine the extent to which established questionnaires have been used.

In line with our preregistered protocol, we synthesised evidence narratively. Meta-analyses could not be undertaken because of the heterogeneity of study designs and outcome measures. We did, however, develop summary tables that include information relating to: sample size, gender composition, mean sample age, cognitive domain, outcome measure, and key findings. Given the range of outcome measures, we attempted to categorise the included studies by common cognitive domains. As the review took an explorative approach, and did not specify domains of interest, categorisation took place after full-text evaluation of included studies.”

e. Item 27: Report which of the following are publicly available and where they can be found: template data collection forms; data extracted from included studies; data used for all analyses; analytic code; any other materials used in the review.

**Author response:** An additional line has been added at the start of the method to reflect that data used to conduct analyses in the review will be publicly available. The revised text can be found on page 6 (line number: 119-122) and reads as follows:

“The systematic review was preregistered at the Open Science Framework (OSF; https://osf.io/uzm5v) as part of a larger study (also assessing the relationships between paranormal beliefs and schizotypal personality traits). Data used for the descriptive and inferential analyses presented in the results section are available at the OSF preregistration.”

3. I like the proposal of the fluid-executive model, but isn’t the general idea that “it is possible to view the association between the many cognitive deficit-paranormal belief associations as the product of a single underlying fluid intelligence-executive component” in conflict with the fact that findings presented in the intelligence section “are highly conflicting, with an almost an equal number of significant versus non-significant findings”?

**Author response:** We have added some text to the summary of the intelligence section to clarify this further. The revised text can be found on page 26 of the revised manuscript (line number: 584-590) and reads as follows:

“Some of this heterogeneity, however, appears to reflect whether studies used crystallised or fluid intelligence tasks and the age of the sample (e.g., Stuart-Hamilton et al [149] failed to find a relationship between fluid IQ and paranormal beliefs in an older sample, but Smith et al. [146] found a significant negative association in a younger sample). The precise relationship of paranormal belief with intelligence requires further investigation, both by considering the age of the sample and assessing relationships with fluid and crystallised intelligence separately.”

4. I think S1 Appendix is not referred in the manuscript.

**Author response:** A line has been added to the end of the “search strategy” section of the method referring to both appendix S1 and S2. The revised text can be found on page 7 of the revised manuscript (line number: 141-143) and reads as follows:

“The PRISMA checklist for abstracts is presented in appendix S1, and the full PRISMA checklist is presented in appendix S2.”

5. Line 97: parenthesis missing

**Author response:** This has been amended.

6. Line 420: I think the role of the beads task in Prike et al.’s study in unclear. In addition, this task is not explained until the next paragraph. Maybe changing the order of those two paragraphs (or explaining the task the first time it is mentioned) would help the reader to understand its relevance.

**Author response:** We have changed the order of these paragraphs so that the description of the task precedes the description of Prike et al.’s study. The revised text can be found on pages 19-20 of the revised manuscript.

7. Line 549: revise “statements”
8. Line 568: revise “be”
9. Line 692: I think “such” is not appropriate there given we have just started a new section

**Author response:** These grammatical revisions have been made.

**Reviewer 2**

1. Line 5, there is a parenthesis missing at the end of the line.

**Author response:** This has been amended.

2. Lines 215-216. Please say explicitly what you mean by large, moderate and small effects.

**Author response:** We have added a statement to clarify these effect sizes (and have provided reference to Cohen’s, 1988, benchmarks to support this). The revised text can be found on page 12 of the revised manuscript (line number: 235-236) and reads as follows:

“…large, moderate and small effects being 0.7, 0.5, and 0.2 respectively [63].”

3. Page 12, first paragraph: any study published as a registered report? This would be interesting because RRs do not only limit p-hacking, they also ensure that that there is no publication bias (papers are accepted or rejected before results are known).

**Author response:** We agree that this would be interesting, however no studies were published as registered reports.

4. Lines 412-414. “Paranormal believers showed a lower perceptual sensitivity compared to sceptics (a bias towards making more ‘yes responses…” Sensitivity and bias are completely different things (e.g., in signal detection theory analysis). Please, clarify whether believers differ in one or the other.

**Author response:** We have amended the text describing these results to reflect the findings more accurately. The revised text can be found on page 19 of the revised manuscript (line number: 407-413) and reads as follows:

“A final study of perceptual decision-making conducted by Van Elk [113] used point-light-walker displays (an animated-point-set of 12 points, representing a human walking on a treadmill), randomly scrambling the location of each individual dot across the display; and participants had to detect if a human agent was present. Paranormal believers were more prone to illusory agency detection than sceptics, being biased towards ‘yes’ responses when no agent was present.”

5. Line 556 “… conducted by similar research teams”. Similar in what sense?

**Author response:** The research teams were “partly overlapping”, with two authors contributing to both studies. This phrase has been removed from the revised manuscript, however, to improve clarity.

**Reviewer 3**

1. The sentence on page 13, lines 263-266, discusses differences between studies with student and non-student samples but the analyses reported do not find significant differences. Please make the lack of significant differences clear and adjust/remove the related discussion.

**Author response:** We have clarified the lack of significant differences in sample size and quality between studies with student and non-student samples. The revised text can be found on page 14 of the revised manuscript (line number 280-282) and reads as follows (statistical tests have been replaced with ellipses here):

“Studies testing undergraduates and non-undergraduates did not differ in mean sample size…or in quality ratings...”

2. When discussing the conjunction fallacy (pages 22 and 25), in addition to Rogers et al., Prike et al. (2017) also found a significant relationship between the conjunction fallacy and paranormal belief. Additionally, the differences between the studies may be due to differences in analysis techniques used. In the Dagnall et al. studies, all the probabilistic reasoning tasks were entered together as predictors, whereas Rogers et al. and Prike et al. looked at the relationship between the conjunction fallacy and paranormal belief without entering/controlling for these other probabilistic reasoning tasks, which may explain the differences in results. This doesn’t need much discussion but may be worth noting or mentioning.

**Author response:** We have included some additional text in the summary for the reasoning section to draw attention to this. The revised text can be found on page 24-25 of the revised manuscript (line number: 544-550) and reads as follows:

“Despite this, evidence regarding the association between paranormal belief and the conjunction fallacy are conflicting, with two studies [129-130] reporting no effect, and three [82, 130-131] reporting significant associations. This may be due, in part, to the different statistical techniques used within each study, as those reporting no effect [129-130] used multiple regression analyses with all probabilistic tasks entered as predictor variables, while studies reporting significant associations [82, 130-131] only included conjunction fallacy tasks in their predictive models.”

3. The section on page 31, lines 701-705, is unnecessarily repetitive and makes the same point multiple times (generally study quality is good but there are some specific areas for improvement).

**Author response:** Much of this text has been deleted to improve clarity. The revised text can be found on pages 29-30 of the revised manuscript.

4. On page 32, line 731, the text says “Eight” but the parentheses say “(9/71)”.

**Author response:** While this text has been removed as part of the above revision, we would like to clarify here that the parentheses were correct (nine papers report positive cognitive correlates of paranormal beliefs) but one study was missed in the in-text description.

**Reviewer 4**

1. On pg 13 (lines 263-266) claims are made that aren’t supported by the provided statistics. So either those statistics are incorrect (or I am misunderstanding what is being reported), or the wording needs to be changed. That is it cannot be said that the undergrad studies tended to have smaller samples and lower quality; however, it could be said that descriptively there looks to be a difference but there is no statistical evidence that there is indeed a difference.

**Author response:** As stated above, the text here has been amended to reflect that these differences were not statistically different. The revised text can be found on page 14 of the revised manuscript (line number 280-282).

2. I appreciate the expanded limitations section, but still think there are issues with it. First, some of the points are underdeveloped; for example, the discussion about weighting vs. summing on the AXIS needs to be unpacked with even just 1-2 more sentences to be clear what specifically the issue may be (pg 120, lines 863-867). Admittedly I am a bit old-school and still believe in the rule that proper paragraphs are a minimum of 3 sentences, but here I am saying it because the 2 sentences that are there do not follow-through the point, especially for readers who may not be familiar with the specific issue.

**Author response:** We have included some additional discussion about weighting vs. summing on the AXIS. The revised text can be found on page 35 of the revised manuscript (line number: 798-801) and reads as follows:

“Thus, two studies with the same total quality score, but across different items, might not be comparable because some items may be more concerning to quality than others. Hence, we have focused on specific domains of strength or weakness across studies.”

3. Further, I expected the issue of categorization to be included in the limitations, so was quite surprised when I saw it was not there. It is mentioned earlier in the manuscript, which was good, but the alternate categories table (S9) shows that it warrants further discussion in terms of limitations. That is, the table very nicely shows just how many potential categories the majority of studies could fall into. So I want to be clear that I am not trying to argue that the review be redone with the studies being included in all possible categories because I understand the author’s justification for the way they decided to do the categorization. However, I do think more consideration is needed for how this effects what we can conclude for each section (i.e., given how many “secondary focus” studies had to be excluded from consideration because they were used elsewhere).

**Author response:** We have included some additional text in the limitations to address this issue. The revised text can be found on 37 of the revised manuscript (line number: 802-809) and reads as follows:

“We acknowledge substantial limitations regarding the classification of studies into six areas of cognitive function: (1) perceptual and cognitive biases, (2) reasoning, (3) intelligence, critical thinking, and academic performance, (4) thinking style, (5) executive function, and (6) other cognitive functions. Table S9 shows that many of the studies could be re-classified and indeed, two-thirds (48/71) could be re-classified as assessing executive functioning. The latter is consistent with our proposal that a substantial proportion of the published studies may be documenting a relationship between paranormal beliefs and higher-level executive function/fluid intelligence.”

We have also stated towards the end of our limitations section (line number: 832-833) that “…conclusions from our systematic review regarding findings for specific cognitive domains must also be interpreted with some caution.”

4. I think the summary sections are quite useful, and go a long way to helping to track through what each section is trying to convey. However, that (along with other additions) does mean that overall the manuscript is a bit daunting to get through, and I think in general it feels like it has a lot of repetition in some spots. For example, the start of the General Discussion is mainly just repetition (including all the numbers again) of what has already been presented. A lot of that could be deleted or streamlined into main points that move beyond what has already been said in earlier sections. And I would also argue focusing on my direct and active writing would cut out a lot of wordiness and also help the manuscript feel more streamlined and manageable (including cutting down on the large amount of information given in parentheses).

**Author response:** Much of the text in this section, and throughout the manuscript, has been deleted or amended to shorten the length of the manuscript and to make the language more active than passive.

5. There is an issue with the wording of the sentence on pg 88, lines 99-102. The first half is hard to follow due to the two “was” near each other, and I think at least one of those needs to be reworked to help clarify what is being said.

**Author response:** This sentence has been reworded to help improve clarity. The revised text can be found on page 5 of the revised manuscript (line number: 107-110), and reads as follows:

“That non-systematic narrative review of 43 studies is now almost 30 years old and may have introduced bias by “…citing null results only when these form a substantial proportion of the available data on a given relationship” (p.6).”

**References:**

Cohen, J. (1988). *Statistical power analysis for the social sciences* (2nd ed). Lawrence Erlbaum Associates.

Mallett, R., Hagen-Zanker, J., Slater, R., & Duvendack, M. (2012). The benefits and challenges of using systematic reviews in international development research. *Journal of development effectiveness, 4*(3), 445-455.
